# Sliding Mode Observer-Based Current Sensor Fault Reconstruction and Unknown Load Disturbance Estimation for PMSM Driven System

**DOI:** 10.3390/s17122833

**Published:** 2017-12-06

**Authors:** Kaihui Zhao, Peng Li, Changfan Zhang, Xiangfei Li, Jing He, Yuliang Lin

**Affiliations:** 1 College of Electrical and Information Engineering, Hunan University of Technology, Zhuzhou 412007, China; zhaokaihui@hut.edu.cn (K.Z.); lipeng@stu.hut.edu.cn (P.L.); zhangchangfan@263.net (C.Z.); lixiangfei2006@163.com (X.L.); 2 College of Mechatronics Engineering and Automation, National University of Defense Technology, Changsha 410000, China; 3 Department of Electrical Engineering, Shandong Polytechnic, Jinan 250104, China; 268@sdp.edu.cn

**Keywords:** permanent magnet synchronous motor (PMSM), sliding mode observers (SMOs), current sensor, fault reconstruction, unknown load disturbance estimation

## Abstract

This paper proposes a new scheme of reconstructing current sensor faults and estimating unknown load disturbance for a permanent magnet synchronous motor (PMSM)-driven system. First, the original PMSM system is transformed into two subsystems; the first subsystem has unknown system load disturbances, which are unrelated to sensor faults, and the second subsystem has sensor faults, but is free from unknown load disturbances. Introducing a new state variable, the augmented subsystem that has sensor faults can be transformed into having actuator faults. Second, two sliding mode observers (SMOs) are designed: the unknown load disturbance is estimated by the first SMO in the subsystem, which has unknown load disturbance, and the sensor faults can be reconstructed using the second SMO in the augmented subsystem, which has sensor faults. The gains of the proposed SMOs and their stability analysis are developed via the solution of linear matrix inequality (LMI). Finally, the effectiveness of the proposed scheme was verified by simulations and experiments. The results demonstrate that the proposed scheme can reconstruct current sensor faults and estimate unknown load disturbance for the PMSM-driven system.

## 1. Introduction

The permanent magnet synchronous motor (PMSM) has been widely used in high-speed trains and electric vehicles, due to its good dynamic response, high torque-to-current ratio, high power density and excellent tracking precision [1]. The reliable operation of PMSM is the basic requirement for the application of high-speed trains and electric vehicles. However, current sensor failure often occurs in the complex PMSM-driven system. The current sensor is located in the feedback channel of the PMSM control system. Even a tiny fault of the current sensor may lead to the misoperation of the PMSM system and destroy the stability of the system. Moreover, the nonlinearities and system disturbances can cause a misleading alarm. Therefore, it is necessary to study the fault diagnosis and fault reconstruction of the PMSM current sensor and improve the overall performance of the PMSM system. This paper proposes a new scheme of reconstructing current sensor faults and estimating unknown load disturbance for the PMSM-driven system.

In recent years, many control methods have been proposed to diagnose the current sensors fault of PMSM. An approach of current sensor fault detection and isolation (FDI) for PMSM drives is presented based on signal analysis instead of currents’ residual generation through observers in [2]. A new approach is proposed for current sensor fault diagnosis of permanent magnetic synchronous generator (PMSG) drives in wind energy conversion systems (WECSs) using the measured phase currents in [3]. In [2,3], it can only detect current fault, but not reconstruct and locate current fault. The method of current sensor fault reconstruction for PMSM drives is presented on the α-β axis in [4]. A current sensor fault reconstruction algorithm for the interior PMSM (IPMSM) system based on active flux linkage by the sliding mode observer is presented in [5,6]. An on-line software fault detection, localization and system reconfiguration method is proposed for PMSM based on the monitoring signals of “abc” currents, DC-link voltage and rotor speed/position in [7]. The current sensor fault diagnosis is presented for the PMSM drive system in [8] based on the differential algebraic method. However, the unknown load disturbance is not considered for the PMSM drive systems in [4,5,6,7,8].

Almost all real dynamic systems can be represented as fully-Lipschitz nonlinear systems, at least locally [9]. The problem of incipient fault diagnosis for a class of Lipschitz nonlinear systems with sensor biases is dealt with by sliding mode observers in [10]. An observer-based sensor fault estimation method for generalized Lipschitz nonlinear systems in the presence of input disturbance and measurement noise is developed in [11]. A sensor fault estimation method is presented for a general class of uncertain Lipschitz nonlinear augmented systems in [12]. A model-based FDI scheme for robot manipulators with actuator and sensor faults is investigated in [13]. State and fault estimations for a class of uncertain Lipschitz nonlinear systems is proposed using a descriptor from the observer and an adaptive sliding mode observer in [14]. The problems of simultaneous actuator and sensor fault detection for a class of uncertain Lipschitz nonlinear systems are considered when the observer matching condition is not satisfied in [15]. Fault reconstruction problems for a class of uncertain Lipschitz nonlinear systems with actuator faults, sensor faults and external disturbances are considered in [16]. An observer-based fault reconstruction method for polymer electrolyte membrane (PEM) fuel cells is presented by the adaptive-gain second-order sliding mode (SOSM) observer in [17]. A high gain observer with multiple sliding modes for simultaneous state and fault estimations for MIMO nonlinear systems is developed in [18]. A higher-order sliding mode observer based on the super-twisting algorithm for state and unknown input estimations is developed for estimating the road adhesion coefficient in [19]. A new scheme for estimating the actuator and sensor fault for Lipschitz nonlinear systems with unstructured uncertainties is proposed using the sliding mode observer technique in [20,21].

Inspired by the above surveys, the PMSM is taken as a Lipschitz nonlinear system. This paper proposes a new scheme of reconstructing current sensor faults and estimating unknown load disturbance for the PMSM-driven system. Two sliding mode observers (SMOs) are designed: the unknown load disturbance is estimated by the first SMO in subsystem, which has unknown load disturbance, and the sensor faults are reconstructed using the second SMO in augmented subsystem which have sensor faults. The gains of the proposed SMOs and their stability analysis are developed via the solution of linear matrix inequality (LMI). The adoption of the LMI algorithm makes it easier to obtain the key parameters of the design of SMOs and relaxes the selection criteria for the PMSM-driven system. The scheme can be applicable for incipient fault, intermittent fault, high frequency and low frequency fault or any other types of faults. This makes the theory of the sliding mode observer applicable to the engineering of PMSM current sensor fault reconstruction.

The remainder of this paper is organized as follows: The system description of PMSM is presented in Section 2, and the model of PMSM is converted into two subsystems. Section 3 designs two SMOs such that the unknown load disturbance is estimated, and the current sensor faults are reconstructed. Stability of the system is proven using Lyapunov analysis. The sufficient conditions for the stability of the scheme are derived and expressed as linear matrix inequalities (LMI). The proposed method is applied to the PMSM systems in Section 4. The overall architecture is tested in simulation and experiment, providing good results. The simulation and experiment results are shown in Section 5. Finally, conclusions are given in Section 6.

## 2. System Description

The dynamic mathematical model of PMSM can be defined in the *d*-*q* reference frame as follows [22]:

(1) $$\left\{\begin{array}{cc} \frac{d\omega_{e}}{dt} & =\frac{3n_{p}^{2}}{2J}\left[\psi_{r}+\left(L_{d}-L_{q}\right)i_{d}\right]i_{q}-\frac{n_{p}}{J}T_{L}-\frac{1}{J}B\omega_{e}\\ \frac{d\theta_{e}}{dt} & =\omega_{e}\\ \frac{di_{q}}{dt} & =-\frac{R_{s}}{L_{q}}i_{q}-\omega_{e}\frac{L_{d}}{L_{q}}i_{d}-\omega_{e}\frac{\psi_{r}}{L_{q}}+\frac{1}{L_{q}}u_{q}\\ \frac{di_{d}}{dt} & =-\frac{R_{s}}{L_{d}}i_{d}+\omega_{e}\frac{L_{q}}{L_{d}}i_{q}+\frac{1}{L_{d}}u_{d}, \end{array}\right.$$

where $R_{s}$ is the stator resistance (Ω); $u_{d}$, $u_{q}$, $i_{d}$, $i_{q}$, $L_{d}$ and $L_{q}$ represent the *d*-*q* axis stator voltages (V), currents (A) and inductances (Wb), respectively; $\psi_{r}$ is the amplitude of the permanent magnet flux linkage (Wb); $\omega_{e}$ and $\theta_{e}$ are the electrical angular velocity (rad/s) and the electrical angle (rad); $n_{p}$ is the number of pole pairs; $T_{L}$ is load torque (N·m); *J* and *B* are the total moment of inertia (kg·m${}^{2}$) and the viscous friction coefficient (Nm·s/rad).

The dynamic mathematical model of IPMSM with current sensors fault can be described as follows:

(2) $$\left\{\begin{array}{c} \underset{\dot{x}}{\underset{︸}{\left[\begin{array}{c} {\dot{\omega }}_{e}\\ {\dot{\theta }}_{e}\\ {\dot{i}}_{q}\\ {\dot{i}}_{d} \end{array}\right]}}=\underset{A}{\underset{︸}{\left[\begin{array}{cccc} -\frac{B}{J} & 0 & \frac{3n_{p}^{2}}{2J}\psi_{r} & 0\\ 1 & 0 & 0 & 0\\ -\frac{\psi_{r}}{L_{q}} & 0 & -\frac{R_{s}}{L_{q}} & 0\\ 0 & 0 & 0 & -\frac{R_{s}}{L_{d}} \end{array}\right]}}\underset{x}{\underset{︸}{\left[\begin{array}{c} \omega_{e}\\ \theta_{e}\\ i_{q}\\ i_{d} \end{array}\right]}}+\underset{B}{\underset{︸}{\left[\begin{array}{cc} 0 & 0\\ 0 & 0\\ \frac{1}{L_{q}} & 0\\ 0 & \frac{1}{L_{d}} \end{array}\right]}}\underset{u}{\underset{︸}{\left[\begin{array}{c} u_{q}\\ u_{d} \end{array}\right]}}+\underset{D}{\underset{︸}{\left[\begin{array}{c} -\frac{n_{p}}{J}\\ 0\\ 0\\ 0 \end{array}\right]}}\underset{d}{\underset{︸}{T_{L}}}+\underset{f(x)}{\underset{︸}{\left[\begin{array}{c} \frac{3n_{p}^{2}\left(L_{d}-L_{q}\right)}{2J}i_{d}i_{q}\\ 0\\ -\frac{L_{d}}{L_{q}}\omega_{e}i_{d}\\ \frac{L_{q}}{L_{d}}\omega_{e}i_{q} \end{array}\right]}}\\ \underset{y}{\underset{︸}{\left[\begin{array}{c} \omega_{e}\\ i_{q}\\ i_{d} \end{array}\right]}}=\underset{C}{\underset{︸}{\left[\begin{array}{cccc} 1 & 0 & 0 & 0\\ 0 & 0 & 1 & 0\\ 0 & 0 & 0 & 1 \end{array}\right]}}\underset{x}{\underset{︸}{\left[\begin{array}{c} \omega_{e}\\ \theta_{e}\\ i_{q}\\ i_{d} \end{array}\right]}}+\underset{E}{\underset{︸}{\left[\begin{array}{cc} 0 & 0\\ 1 & 0\\ 0 & 1 \end{array}\right]}}\underset{f_{s}}{\underset{︸}{\left[\begin{array}{c} f_{q}\\ f_{d} \end{array}\right]}}, \end{array}\right.$$

where $f_{q}$ and $f_{d}$ denote the current sensor fault of the *q*-axis and *d*-axis.

Equation (2) can be transformed into the following nonlinear system:

(3) $$\left\{\begin{array}{cc} \dot{x}\left(t\right) & =\mathrm{Ax}(t)+f(x,t)+\mathrm{Bu}(t)+\mathrm{Dd}(t)\\ y(t) & =\mathrm{Cx}\left(t\right)+{\mathrm{Ef}}_{s}\left(t\right), \end{array}\right.$$

where $x\left(t\right)\in R^{n}$, $x={\left[\begin{array}{cccc} \omega_{e} & \theta_{e} & i_{q} & i_{d} \end{array}\right]}^{T}$ is the state vector; $u\left(t\right)\in R^{m}$, $u={\left[\begin{array}{cc} u_{q} & u_{d} \end{array}\right]}^{T}$ is the measurable input vector; $y\left(t\right)\in R^{p}$, $y={\left[\begin{array}{ccc} \omega_{e} & i_{q} & i_{d} \end{array}\right]}^{T}$ is the measurable output vector; $d\left(t\right)\in R^{r}$, $d=T_{L}$ is unknown input disturbances; $f_{s}\left(t\right)\in R^{q}$, $f_{s}\left(t\right)=\left[\begin{array}{cc} f_{q} & f_{d} \end{array}\right]$ denote the sensor faults, which is unknown bounded; $A\in R^{n\times n}$, $B\in R^{n\times m}$ and $C\in R^{p\times n}$ are known matrices; $D\in R^{n\times r}$ is the known corresponding disturbance distribution matrix; $E\in R^{p\times q}$ is the known distribution matrix of sensor faults; $f\left(x,t\right)\in R^{n}$ is the known nonlinear function. For System (2), n=4, m=2, p=3, q=2, r=1 and p≥q+r.

**Proposition** **1.** *The known nonlinear term f(x,t) is assumed to satisfy the Lipschitz condition:*

$$∥f\left(x,t\right)-f\left(\hat{x},t\right)∥\leq \gamma ∥x-\hat{x}∥.$$

**Proposition** **2.** The function d denotes the unknown input disturbances, but bounded, and it satisfies $∥d∥\leq \rho_{d}$. Furthermore, the unknown sensor fault $f_{s}$ is bounded, and it satisfies $∥f_{s}∥\leq \rho_{s}$.

**Proposition** **3.** C and D are full column rank, and rank(CD)=rank(D)=r.

If Assumption 3 holds, there are two transformation nonsingular matrices *T* and *S* [23]. The original System (3) can be transformed into the following two subsystems.

(4) $$\begin{array}{c} \left\{\begin{array}{cc} {\dot{x}}_{1} & =A_{11}x_{1}+A_{12}x_{2}+B_{1}u+f_{1}\left(x,t\right)+D_{1}d\\ y_{1} & =C_{11}x_{1}, \end{array}\right. \end{array}$$

(5) $$\begin{array}{c} \left\{\begin{array}{cc} {\dot{x}}_{2} & =A_{21}x_{1}+A_{22}x_{2}+B_{2}u+f_{2}\left(x,t\right)\\ y_{2} & =C_{22}x_{2}+E_{2}f_{s}, \end{array}\right. \end{array}$$

where $x={\left[\begin{array}{cc} x_{1} & x_{2} \end{array}\right]}^{T}$, $x_{1}\in R^{r}$, $x_{2}\in R^{n-r}$; $y={\left[\begin{array}{cc} y_{1} & y_{2} \end{array}\right]}^{T}$, $y_{1}\in R^{r}$, $y_{2}\in R^{p-r}$. $A_{11}\in R^{r\times r}$, $A_{12}\in R^{r\times (n-r)}$, $A_{21}\in R^{(n-r)\times r}$, $A_{22}\in R^{\left(n-r)\times (n-r\right)}$, $B_{1}\in R^{r\times m}$, $B_{2}\in R^{(n-r)\times m}$, $C_{11}\in R^{r\times r}$, $C_{22}\in R^{\left(p-r)\times (n-r\right)}$, $D_{1}\in R^{r\times r}$, $E_{2}\in R^{(p-r)\times q}$, $f_{1}\in R^{r}$,$f_{2}\in R^{n-r}$.

The matrix transformation in Systems (4) and (5) is as follows:

$${\mathrm{TAT}}^{-1}=\left[\begin{array}{cc} A_{11} & A_{12}\\ A_{21} & A_{22} \end{array}\right],\mathrm{TB}=\left[\begin{array}{c} B_{1}\\ B_{2} \end{array}\right],\mathrm{TD}=\left[\begin{array}{c} D_{1}\\ 0 \end{array}\right],\mathrm{SE}=\left[\begin{array}{c} 0\\ E_{2} \end{array}\right],{\mathrm{SCT}}^{-1}=\left[\begin{array}{cc} C_{11} & 0\\ 0 & C_{22} \end{array}\right].$$

where $C_{11}$ and $D_{1}$ are invertible matrices, and $rank(D_{1})=r$.

**Remark** **1.** Subsystem (4) contains only unknown disturbances d, without sensor fault $f_{s}$; while Subsystem (5) has only sensor faults $f_{s}$, without unknown disturbances d. The sensor faults $f_{s}$ and the unknown disturbance d can be completely decoupled in the new systems.

For Subsystem (5), define a new state $x_{3}=\int_{0}^{t}y_{2}\left(\tau \right)d\tau$, so that:

(6) $${\dot{x}}_{3}=A_{s}x_{3}+B_{s}y_{2},$$

where $x_{3}\in R^{p-r}$ is the new state vector; $A_{s}$, $B_{s}$ are the filter matrices to be designed, $A_{s}\in R^{\left(p-r)\times (p-r\right)}$, $A_{s}$ is an arbitrary filter matrix; $B_{s}\in R^{\left(p-r)\times (p-r\right)}$, $B_{s}$ is a full rank constant matrix.

If $A_{s}$ is chosen as the zero matrix and $B_{s}$ is chosen as the identity matrix, substituting Equation (5) into Equation (6), Equation (6) can now be expressed as follows:

(7) $${\dot{x}}_{3}=C_{22}x_{2}+E_{2}f_{s}.$$

Based on Equation (5) and (7), the augmented new subsystem can be expressed as follows:

(8) $$\left\{\begin{array}{cc} \left[\begin{array}{c} {\dot{x}}_{2}\\ {\dot{x}}_{3} \end{array}\right] & =\left[\begin{array}{cc} A_{22} & 0\\ C_{22} & 0 \end{array}\right]\left[\begin{array}{c} x_{2}\\ x_{3} \end{array}\right]+\left[\begin{array}{c} A_{21}\\ 0 \end{array}\right]x_{1}+\left[\begin{array}{c} f_{2}\left(x,t\right)\\ 0 \end{array}\right]+\left[\begin{array}{c} B_{2}\\ 0 \end{array}\right]u+\left[\begin{array}{c} 0\\ E_{2} \end{array}\right]f_{s}\\ x_{3} & =\left[\begin{array}{cc} 0 & I \end{array}\right]\left[\begin{array}{c} x_{2}\\ x_{3} \end{array}\right]. \end{array}\right.$$

The augmented System (8) can then be rewritten in a more compact form as:

(9) $$\left\{\begin{array}{cc} {\dot{x}}_{0} & =A_{0}x_{0}+A_{1}x_{1}+F_{2}\left(x,t\right)+B_{0}u+E_{0}f_{s}\\ y_{0} & =C_{0}x_{0}, \end{array}\right.$$

where $x_{0}={\left[\begin{array}{cc} x_{2} & x_{3} \end{array}\right]}^{T}$, $y_{0}=x_{3}$; $x_{0}\in R^{n+p-2r}$, $y_{0}\in R^{p-r}$, $A_{0}=\left[\begin{array}{cc} A_{22} & 0\\ C_{22} & 0 \end{array}\right]$, $A_{1}=\left[\begin{array}{c} A_{21}\\ 0 \end{array}\right]$, $F_{2}=\left[\begin{array}{c} f_{2}\left(x,t\right)\\ 0 \end{array}\right]$, $B_{0}=\left[\begin{array}{c} B_{2}\\ 0 \end{array}\right]$, $E_{0}=\left[\begin{array}{c} 0\\ E_{2} \end{array}\right]$, $C_{0}=\left[\begin{array}{cc} 0 & I_{p-r} \end{array}\right]$. $A_{0}\in R^{\left(n+p-2r)\times (n+p-2r\right)}$, $A_{1}\in R^{(n+p-2r)\times r}$, $B_{0}\in R^{(n+p-2r)\times m}$, $C_{0}\in R^{\left(p-r)\times (n+p-2r\right)}$, $E_{0}\in R^{(n+p-2r)\times q}$, $F_{2}\in R^{n+p-2r}$.

Accordingly, Subsystem (4) is rewritten as:

(10) $$\left\{\begin{array}{cc} {\dot{x}}_{1} & =A_{11}x_{1}+A_{2}x_{0}+B_{1}u+f_{1}\left(x,t\right)+D_{1}d\\ y_{1} & =C_{11}x_{1}, \end{array}\right.$$

where $A_{2}=\left[\begin{array}{cc} A_{12} & 0_{r\times (p-r)} \end{array}\right]$, $A_{11}\in R^{r\times r}$, $A_{12}\in R^{r\times (n-r)}$, $A_{2}\in R^{r\times (n+p-2r)}$, $C_{11}\in R^{r\times r}$, $B_{1}\in R^{r\times m}$, $D_{1}\in R^{r\times r}$, $f_{1}\in R^{r}$, $y_{1}\in R^{r}$.

**Remark** **2.** Subsystem (4) can be changed to the augmented System (10). Subsystem (5) can be changed to the augmented System (9), which shows that the sensor fault is converted to equivalent pseudo actuator fault by introducing state variable $x_{3}$. Then, the sensor fault reconstruction can be realized with the method of actuator fault reconstruction.

## 3. Sensors’ Fault Reconstruction and Unknown Disturbance Estimation Using Sliding Mode Observers

In this section, two sliding mode observers are designed for Subsystem (9) and Subsystem (10) respectively; one of which is to reconstruct the sensor fault, while the other one is to estimate the unknown disturbance. The simultaneous reconstruction of the sensor faults and estimation of the unknown disturbance are studied.

### 3.1. Sliding Mode Observers Design

**Proposition** **4.** *The nonlinear term $f_{1}\left(x,t\right)$, F(x,t) satisfies the Lipschitz condition,*

(11) $$∥f_{1}\left(x,t\right)-f_{1}\left(\hat{x},t\right)∥\leq \gamma_{1}∥x-\hat{x}∥,$$

(12) $$∥F\left(x,t\right)-F\left(\hat{x},t\right)∥=∥f_{2}\left(x,t\right)-f_{2}\left(\hat{x},t\right)∥\leq \gamma_{0}∥x-\hat{x}∥,$$

*where $\gamma_{1}$, $\gamma_{0}$ are Lipschitz constants.*

**Proposition** **5.** *For every complex number s with nonnegative real part [21]:*

(13) $$rank\left[\begin{array}{cc} sI_{n}-A & D\\ C & 0 \end{array}\right]=n+rank\left(D\right).$$

*This is known as the minimum phase condition.*

**Lemma** **1.** *If Assumption 5 holds, then the pairs $\left(A_{11},C_{11}\right)$ and $\left(A_{0},C_{0}\right)$ are observable [23], there exist matrices $L_{0}$ and $L_{1}$, such that $A_{01}=A_{11}-L_{1}C_{11}$ and $A_{00}=A_{0}-L_{0}C_{0}$ are stable and the following Lyapunov equations hold:*

(14) $$A_{00}^{T}P_{0}+P_{0}A_{00}=-Q_{0},$$

(15) $$A_{01}^{T}P_{1}+P_{1}A_{01}=-Q_{1},$$

*where $P_{1}$, $Q_{1}$, $P_{0}$, $Q_{0}$ are all symmetric positive definite (SPD) matrices.*

**Proposition** **6.** *There exists an arbitrary matrix $F_{0}\in R^{\left(n+q-2r)\times (n+q-2r\right)}$, $F_{1}\in R^{r\times r}$, such that:*

(16) $$P_{0}E_{0}=C_{0}^{T}F_{0}^{T},$$

(17) $$P_{1}D_{1}=C_{11}^{T}F_{1}^{T},$$

*where $C_{0}\in R^{\left(p-r)\times (n+p-2r\right)}$, $E_{0}\in R^{(n+p-2r)\times q}$, $D_{1}\in R^{r\times r}$, $C_{11}\in R^{r\times r}$.*

For Subsystem (10), the proposed sliding mode observer is constructed:

(18) $$\left\{\begin{array}{cc} {\dot{\hat{x}}}_{1} & =A_{11}{\hat{x}}_{1}+A_{2}{\hat{x}}_{0}+B_{1}u+f_{1}\left(\hat{x},t\right)+D_{1}w_{1}+L_{1}\left(y_{1}-{\hat{y}}_{1}\right)\\ {\hat{y}}_{1} & =C_{11}{\hat{x}}_{1}, \end{array}\right.$$

where $w_{1}$ is the input control signal of the sliding mode, defined by:

(19) $$w_{1}=\left\{\begin{array}{cc} -\rho_{1}\frac{F_{1}\left({\hat{y}}_{1}-y_{1}\right)}{∥F_{1}\left({\hat{y}}_{1}-y_{1}\right)∥}\; & if\;{\hat{y}}_{1}-y_{1}\neq 0\\ 0\; & if\;{\hat{y}}_{1}-y_{1}=0, \end{array}\right.$$

where $F_{1}\in R^{r\times r}$ is the matrix to be designed, $\rho_{1}$ is the scalar function to be designed and $\rho_{1}\geq \rho_{d}$.

For Subsystem (9), the proposed sliding mode observer is constructed:

(20) $$\left\{\begin{array}{cc} {\dot{\hat{x}}}_{0} & =A_{0}{\hat{x}}_{0}+A_{1}{\hat{x}}_{1}+F_{2}\left(\hat{x},t\right)+B_{0}u+E_{0}w_{0}+L_{0}\left(y_{0}-{\hat{y}}_{0}\right)\\ {\hat{y}}_{0} & =C_{0}{\hat{x}}_{0}, \end{array}\right.$$

where $w_{0}$ is the input control signal of the sliding mode, defined by:

(21) $$w_{0}=\left\{\begin{array}{cc} -\rho_{0}\frac{F_{0}\left({\hat{y}}_{0}-y_{0}\right)}{∥F_{0}\left({\hat{y}}_{0}-y_{0}\right)∥}\; & if\;{\hat{y}}_{0}-y_{0}\neq 0\\ 0\; & if\;{\hat{y}}_{0}-y_{0}=0, \end{array}\right.$$

where $F_{0}\in R^{\left(n+q-2r)\times (n+q-2r\right)}$ is the matrix to be designed, $\rho_{0}$ is the scalar function to be designed and $\rho_{0}\geq \rho_{s}$.

The state estimation errors are defined as:

(22) $$e={\left[\begin{array}{cc} e_{1} & e_{0} \end{array}\right]}^{T},$$

where $e_{1}=x_{1}-{\hat{x}}_{1}$, $e_{0}=x_{0}-{\hat{x}}_{0}$.

The output estimation errors are as follows:

(23) $$\begin{array}{c} e_{y_{1}}=y_{1}-{\hat{y}}_{1}=C_{11}e_{1}\\ e_{y_{0}}=y_{0}-{\hat{y}}_{0}=C_{0}e_{0}. \end{array}$$

Based on Equations (9), (10), (18) and (20), the corresponding error dynamic equations are given by:

(24) $$\begin{array}{cc} {\dot{e}}_{1} & =\left(A_{11}-L_{1}C_{11}\right)e_{1}+A_{2}e_{0}+f_{1}\left(x,t\right)-f_{1}\left(\hat{x},t\right)+D_{1}\left(d-w_{1}\right)\\ & =A_{01}e_{1}+A_{2}e_{0}+f_{1}\left(x,t\right)-f_{1}\left(\hat{x},t\right)+D_{1}\left(d-w_{1}\right), \end{array}$$

(25) $$\begin{array}{cc} {\dot{e}}_{0} & =\left(A_{0}-L_{0}C_{0}\right)e_{0}+F_{2}\left(x,t\right)-F_{2}\left(\hat{x},t\right)+E_{0}\left(f_{s}-w_{0}\right)\\ & =A_{00}e_{0}+A_{1}e_{1}+F_{2}\left(x,t\right)-F_{2}\left(\hat{x},t\right)+E_{0}\left(f_{s}-w_{0}\right). \end{array}$$

Define the sliding mode surface as:

(26) $$s=\{\left.\left(e_{1},e_{0}\right)\right|e_{1}=0,e_{0}=0\}.$$

**Lemma** **2** (Schur complement [24])**.** *For a given symmetric matrix $S=\left[\begin{array}{cc} S_{11} & S_{12}\\ S_{21} & S_{22} \end{array}\right]$, where $S_{11}\in R^{r\times r}$, the following three conditions are equivalent:*

*1.*  S<0;
*2.*  $S_{11}<0$, $S_{22}-S_{12}^{T}S_{11}^{-1}S_{12}<0$;
*3.*  $S_{22}<0$, $S_{11}-S_{12}S_{22}^{-1}S_{12}^{T}<0$.

**Lemma** **3** ([25])**.** *If g(x,u,t) satisfies the Lipschitz condition, there is a symmetric positive definite matrix P that satisfies the following equation:*

(27) $$2\varepsilon^{T}P\left(g\left(x_{1},u,t\right)-g\left(x_{2},u,t\right)\right)\leq k^{2}\varepsilon^{T}\mathrm{PP}\varepsilon +\varepsilon^{T}\varepsilon ,$$

*where $\varepsilon =x_{1}-x_{2}$, k is the Lipschitz constant.*

### 3.2. Lyapunov Stability Analysis

**Theorem** **1.** *Under Assumptions 1–5, if the following LMI holds,*

(28) $$\left[\begin{array}{cccc} H_{1} & P_{1} & A_{1}^{T}P_{0}+P_{1}A_{2} & 0\\ P_{1} & -1/\gamma_{1}^{2} & 0 & 0\\ A_{2}^{T}P_{1}+P_{0}A_{1} & 0 & H_{2} & P_{0}\\ 0 & 0 & P_{0} & 1/\gamma_{0}^{2} \end{array}\right]<0,$$

*where $H_{1}=A_{11}^{T}P_{1}+P_{1}A_{11}-Y_{1}^{T}-Y_{1}+I_{r}$, $H_{2}=A_{0}^{T}P_{0}+P_{0}A_{0}-Y_{0}^{T}-Y_{0}+I_{n+p-2r}$, $Y_{1}=P_{1}L_{1}C_{11}$, $Y_{0}=P_{0}L_{0}C_{0}$, if there exist matrices $P_{1}>0$, $P_{0}>0$, then the error dynamical Systems (24) and (25) are asymptotically stable, and $e_{0}$, $e_{1}$ will converge to the zero point in finite time.*

**Proof** **of** **Theorem** **1.** Consider the following Lyapunov function:

(29) $$V=V_{1}+V_{0}=e_{1}^{T}P_{1}e_{1}+e_{0}^{T}P_{0}e_{0},$$

where $V_{1}=e_{1}^{T}P_{1}e_{1}$, $V_{0}=e_{0}^{T}P_{0}e_{0}$.

The derivative of the Lyapunov function $V_{1}$ with respect to time is:

(30) $$\begin{array}{ccc} {\dot{V}}_{1} & = & {\dot{e}}_{1}^{T}P_{1}e_{1}+e_{1}^{T}P_{1}{\dot{e}}_{1}\\ & = & {\left[A_{01}e_{1}+A_{2}e_{0}+f_{1}\left(x,t\right)-f_{1}\left(\hat{x},t\right)+D_{1}\left(d-w_{1}\right)\right]}^{T}P_{1}e_{1}\\ & & +e_{1}^{T}P_{1}\left[A_{01}e_{1}+A_{2}e_{0}+f_{1}\left(x,t\right)-f_{1}\left(\hat{x},t\right)+D_{1}\left(d-w_{1}\right)\right]\\ & = & e_{1}^{T}\left(A_{01}^{T}P_{1}+P_{1}A_{01}\right)e_{1}+2e_{1}^{T}P_{1}A_{2}e_{0}+2e_{1}^{T}P_{1}\left[f_{1}\left(x,t\right)-f_{1}\left(\hat{x},t\right)\right]\\ & & +2e_{1}^{T}P_{1}D_{1}\left(d-w_{1}\right). \end{array}$$

Similarly, the derivative of $V_{0}$ can be obtained as:

(31) $$\begin{array}{ccc} {\dot{V}}_{0} & = & {\dot{e}}_{0}^{T}P_{0}e_{0}+e_{0}^{T}P_{0}{\dot{e}}_{0}\\ & = & {\left[A_{00}e_{0}+A_{1}e_{1}+F_{2}\left(x,t\right)-F_{2}\left(\hat{x},t\right)+E_{0}\left(f_{s}-w_{0}\right)\right]}^{T}P_{0}e_{0}\\ & & +e_{0}^{T}P_{0}\left[A_{00}e_{0}+A_{1}e_{1}+F_{2}\left(x,t\right)-F_{2}\left(\hat{x},t\right)+E_{0}\left(f_{s}-w_{0}\right)\right]\\ & = & e_{0}^{T}\left(A_{00}^{T}P_{0}+P_{0}A_{00}\right)e_{0}+2e_{0}^{T}P_{0}A_{1}e_{1}+2e_{0}^{T}P_{0}\left[F_{2}\left(x,t\right)-F_{2}\left(\hat{x},t\right)\right]\\ & & +2e_{0}^{T}P_{0}E_{0}\left(f_{s}-w_{0}\right). \end{array}$$

Combining (30) and (31) yields:

(32) $$\begin{array}{cc} \dot{V} & =e_{1}^{T}\left(A_{01}^{T}P_{1}+P_{1}A_{01}\right)e_{1}+2e_{1}^{T}P_{1}A_{2}e_{0}+2e_{1}^{T}P_{1}\left[f_{1}\left(x,t\right)-f_{1}\left(\hat{x},t\right)\right]\\ & +2e_{1}^{T}P_{1}D_{1}\left(d-w_{1}\right)+e_{0}^{T}\left(A_{00}^{T}P_{0}+P_{0}A_{00}\right)e_{0}+2e_{0}^{T}P_{0}A_{1}e_{1}\\ & +2e_{0}^{T}P_{0}\left[F_{2}\left(x,t\right)-F_{2}\left(\hat{x},t\right)\right]+2e_{0}^{T}P_{0}E_{0}\left(f_{s}-w_{0}\right). \end{array}$$

It is easy to see that from Propositions 2 and 5:

(33) $$\begin{array}{c} e_{1}^{T}P_{1}D_{1}\left(d-w_{1}\right)=e_{1}^{T}{\bar{C}}_{11}^{T}F_{1}^{T}d-e_{1}^{T}{\bar{C}}_{11}^{T}F_{1}^{T}w_{1}\\ =F_{1}e_{y1}d-\rho_{1}F_{1}e_{y1}\frac{F_{1}e_{y1}}{∥F_{1}e_{y1}∥}\leq ∥F_{1}e_{y1}∥\left(\rho_{d}-\rho_{1}\right)\leq 0, \end{array}$$

(34) $$\begin{array}{c} e_{0}^{T}P_{0}E_{0}\left(f_{s}-w_{0}\right)=e_{0}^{T}C_{0}^{T}F_{0}^{T}f_{s}-e_{0}^{T}C_{0}^{T}F_{0}^{T}w_{0}\\ =F_{0}e_{y0}f_{s}-\rho_{0}F_{0}e_{y0}\frac{F_{0}e_{y0}}{∥F_{0}e_{y0}∥}\leq ∥F_{0}e_{y0}∥\left(\rho_{s}-\rho_{0}\right)\leq 0. \end{array}$$

From Lemma 3, we find that:

(35) $$2e_{1}^{T}P_{1}\left[f_{1}\left(x,t\right)-f_{1}\left(\hat{x},t\right)\right]\leq \gamma_{1}^{2}e_{1}^{T}P_{1}P_{1}e_{1}+e_{1}^{T}e_{1},$$

(36) $$2e_{0}^{T}P_{0}\left[F\left(x,t\right)-F\left(\hat{x},t\right)\right]\leq \gamma_{0}^{2}e_{0}^{T}P_{0}P_{0}e_{0}+e_{0}^{T}e_{0}.$$

Combining (33)–(36) yields:

(37) $$\begin{array}{cc} \dot{V} & \leq e_{1}^{T}\left(A_{01}^{T}P_{1}+P_{1}A_{01}\right)e_{1}+2e_{1}^{T}P_{1}A_{2}e_{0}+\gamma_{1}^{2}e_{1}^{T}P_{1}P_{1}e_{1}+e_{1}^{T}e_{1}\\ & +e_{0}^{T}\left(A_{00}^{T}P_{0}+P_{0}A_{00}\right)e_{0}+2e_{0}^{T}P_{0}A_{1}e_{1}+\gamma_{0}^{2}e_{0}^{T}P_{0}P_{0}e_{0}+e_{0}^{T}e_{0}\\ & \leq {\left[\begin{array}{c} e_{1}\\ e_{0} \end{array}\right]}^{T}\left[\begin{array}{cc} A_{01}^{T}P_{1}+P_{1}A_{01}+\gamma_{1}^{2}P_{1}P_{1}+I & P_{1}A_{2}+A_{1}^{T}P_{0}\\ A_{2}^{T}P_{1}+P_{0}A_{1} & A_{00}^{T}P_{0}+P_{0}A_{00}+\gamma_{0}^{2}P_{0}P_{0}+I \end{array}\right]\left[\begin{array}{c} e_{1}\\ e_{0} \end{array}\right]. \end{array}$$

To satisfied V<0, it follows that:

(38) $$\left[\begin{array}{cc} A_{01}^{T}P_{1}+P_{1}A_{01}+\gamma_{1}^{2}P_{1}P_{1}+I & P_{1}A_{2}+A_{1}^{T}P_{0}\\ A_{2}^{T}P_{1}+P_{0}A_{1} & A_{00}^{T}P_{0}+P_{0}A_{00}+\gamma_{0}^{2}P_{0}P_{0}+I \end{array}\right]<0.$$

Then, Inequality (38) can be transformed into the following LMI feasibility problem:

(39) $$\left[\begin{array}{cccc} H_{1} & P_{1} & A_{1}^{T}P_{0}+P_{1}A_{2} & 0\\ P_{1} & -1/\gamma_{1}^{2} & 0 & 0\\ A_{2}^{T}P_{1}+P_{0}A_{1} & 0 & H_{2} & P_{0}\\ 0 & 0 & P_{0} & 1/\gamma_{0}^{2} \end{array}\right]<0,$$

where $H_{1}=A_{11}^{T}P_{1}+P_{1}A_{11}-Y_{1}^{T}-Y_{1}+I_{r}$, $H_{2}=A_{0}^{T}P_{0}+P_{0}A_{0}-Y_{0}^{T}-Y_{0}+I_{n+p-2r}$, $Y_{1}=P_{1}L_{1}C_{11}$, $Y_{0}=P_{0}L_{0}C_{0}$.

Then, the observer error dynamics (24) and (25) is asymptotically stable, and $e_{0}$, $e_{1}$ will converge to the zero point in finite time.

This completes the proof. ☐

### 3.3. Sensor Fault Reconstruction and Unknown Load Disturbance Estimation

After a finite period of time, the system state reaches the sliding surface. According to the sliding mode equivalence principle [26], the following equations are obtained:

(40) $$\left\{\begin{array}{cc} e_{1} & ={\dot{e}}_{1}=0\\ e_{0} & ={\dot{e}}_{0}=0, \end{array}\right.$$

Substituting Equation (40) into error dynamics Equations (24) and (25) yields:

(41) $$\begin{array}{cc} 0 & =\left[f_{1}\left(x,t\right)-f_{1}\left(\hat{x},t\right)\right]+D_{1}\left(d-w_{1eq}\right), \end{array}$$

(42) $$\begin{array}{cc} 0 & =\left[F_{2}\left(x,t\right)-F_{2}\left(\hat{x},t\right)\right]+E_{0}\left(f_{s}-w_{0eq}\right). \end{array}$$

Since $\lim_{t\to \infty }e\left(t\right)=0$, $\lim_{t\to \infty }\left[F\left(x,t\right)-F\left(\hat{x},t\right)\right]=0$, $\lim_{t\to \infty }\left[f_{1}\left(x,t\right)-f_{1}\left(\hat{x},t\right)\right]=0$, it follows from Equations (41) and (42) that:

(43) $$\left\{\begin{array}{c} d\to w_{1eq}\\ f_{s}\to w_{0eq}, \end{array}\right.$$

where $w_{1eq}$, $w_{0eq}$ can be approximated as:

(44) $$\left\{\begin{array}{cc} w_{1eq} & =-\rho_{1}\frac{F_{1}\left({\hat{y}}_{1}-y_{1}\right)}{∥F_{1}\left({\hat{y}}_{1}-y_{1}\right)∥+\delta }\\ w_{0eq} & =-\rho_{0}\frac{F_{0}\left({\hat{y}}_{0}-y_{0}\right)}{∥F_{0}\left({\hat{y}}_{0}-y_{0}\right)∥+\delta }, \end{array}\right.$$

where δ is a small positive constant to reduce the chattering effect. It can obtain a smooth fault and disturbance reconstruction.

The unknown input disturbance d and sensor faults $f_{s}$ can be estimated as:

(45) $$\left\{\begin{array}{c} \hat{d}=-\rho_{1}\frac{F_{1}\left({\hat{y}}_{1}-y_{1}\right)}{∥F_{1}\left({\hat{y}}_{1}-y_{1}\right)∥+\delta }\\ {\hat{f}}_{s}=-\rho_{0}\frac{F_{0}\left({\hat{y}}_{0}-y_{0}\right)}{∥F_{0}\left({\hat{y}}_{0}-y_{0}\right)∥+\delta }. \end{array}\right.$$

## 4. Example: Reconstruct Current Sensor Faults and Estimate the Unknown Load for PMSM

In this section, taking the PMSM drive system as an example, the effectiveness of the scheme in sensor fault reconstruction and unknown load estimation is demonstrated. The nonsingular transformation matrices *T* and *S* are chosen as:

$$T=\left[\begin{array}{cccc} 1 & 0 & -1 & -1\\ 0 & 1 & 0 & 0\\ 0 & 0 & 1 & 0\\ 0 & 0 & 0 & 1 \end{array}\right],S=\left[\begin{array}{ccc} 1 & -1 & -1\\ 0 & 1 & 0\\ 0 & 0 & 1 \end{array}\right],$$

then, the PMSM System (2) can be converted into the following two subsystems:

(46) $$\begin{array}{c} \left\{\begin{array}{c} \underset{{\dot{x}}_{1}}{\underset{︸}{{\dot{\omega }}_{e}}}=\underset{A_{11}}{\underset{︸}{\left[-\frac{B}{J}\right]}}\underset{x_{1}}{\underset{︸}{\omega_{e}}}+\underset{A_{12}}{\underset{︸}{\left[\begin{array}{ccc} 0 & \frac{3n_{p}^{2}}{2J}\psi_{r} & 0 \end{array}\right]}}\underset{x_{2}}{\underset{︸}{\left[\begin{array}{c} \theta_{e}\\ i_{q}\\ i_{d} \end{array}\right]}}+\underset{f_{1}\left(x\right)}{\underset{︸}{\left(\frac{3n_{p}^{2}\left(L_{d}-L_{q}\right)}{2J}i_{d}i_{q}\right)}}+\underset{D_{1}}{\underset{︸}{\left[-\frac{n_{p}}{J}\right]}}\underset{d}{\underset{︸}{T_{L}}}\\ \underset{y_{1}}{\underset{︸}{\omega_{e}}}=\underset{C_{11}}{\underset{︸}{1}}\cdot \underset{x_{1}}{\underset{︸}{\omega_{e}}}, \end{array}\right. \end{array}$$

(47) $$\begin{array}{c} \left\{\begin{array}{c} \underset{{\dot{x}}_{2}}{\underset{︸}{\left[\begin{array}{c} {\dot{\theta }}_{e}\\ {\dot{i}}_{q}\\ {\dot{i}}_{d} \end{array}\right]}}=\underset{A_{21}}{\underset{︸}{\left[\begin{array}{c} 1\\ -\frac{\psi_{r}}{L_{q}}\\ 0 \end{array}\right]}}\underset{x_{1}}{\underset{︸}{\omega_{e}}}+\underset{A_{22}}{\underset{︸}{\left[\begin{array}{ccc} 0 & 0 & 0\\ 0 & -\frac{R_{s}}{L_{q}} & 0\\ 0 & 0 & -\frac{R_{s}}{L_{d}} \end{array}\right]}}\underset{x_{2}}{\underset{︸}{\left[\begin{array}{c} \theta_{e}\\ i_{q}\\ i_{d} \end{array}\right]}}+\underset{B_{2}}{\underset{︸}{\left[\begin{array}{cc} 0 & 0\\ \frac{1}{L_{q}} & 0\\ 0 & \frac{1}{L_{d}} \end{array}\right]}}\underset{u}{\underset{︸}{\left[\begin{array}{c} u_{q}\\ u_{d} \end{array}\right]}}+\underset{f_{2}\left(x\right)}{\underset{︸}{\left[\begin{array}{c} 0\\ -\frac{L_{d}}{L_{q}}\omega_{e}i_{d}\\ \frac{L_{q}}{L_{d}}\omega_{e}i_{q} \end{array}\right]}}\\ \underset{y_{2}}{\underset{︸}{\left[\begin{array}{c} i_{q}\\ i_{d} \end{array}\right]}}=\underset{C_{22}}{\underset{︸}{\left[\begin{array}{ccc} 0 & 1 & 0\\ 0 & 0 & 1 \end{array}\right]}}\underset{x_{2}}{\underset{︸}{\left[\begin{array}{c} \theta_{e}\\ i_{q}\\ i_{d} \end{array}\right]}}+\underset{E_{2}}{\underset{︸}{\left[\begin{array}{cc} 1 & 0\\ 0 & 1 \end{array}\right]}}\underset{f_{s}}{\underset{︸}{\left[\begin{array}{c} f_{q}\\ f_{d} \end{array}\right]}}. \end{array}\right. \end{array}$$

For Subsystem (47), a new state $x_{3}=\int_{0}^{t}y_{2}\left(\tau \right)d\tau$ is defined, and $A_{s}=0$ and $B_{s}=I$ are chosen; it can be obtained from Equation (7):

(48) $${\dot{x}}_{3}=C_{22}x_{2}+E_{2}f_{s}=\underset{C_{22}}{\underset{︸}{\left[\begin{array}{ccc} 0 & 1 & 0\\ 0 & 0 & 1 \end{array}\right]}}\underset{x_{2}}{\underset{︸}{\left[\begin{array}{c} \theta_{e}\\ i_{q}\\ i_{d} \end{array}\right]}}+\underset{E_{2}}{\underset{︸}{\left[\begin{array}{cc} 1 & 0\\ 0 & 1 \end{array}\right]}}\underset{f_{s}}{\underset{︸}{\left[\begin{array}{c} f_{q}\\ f_{d} \end{array}\right]}}=\left[\begin{array}{c} i_{q}+f_{q}\\ i_{d}+f_{d} \end{array}\right].$$

From Equation (7), it is easy to see:

(49) $$\begin{array}{c} \left\{\begin{array}{cc} x_{0} & ={\left[\begin{array}{cc} x_{2} & x_{3} \end{array}\right]}^{T}={\left[\begin{array}{ccccc} \theta_{e} & i_{q} & i_{d} & \int \left(i_{q}+f_{q}\right)dt & \int \left(i_{d}+f_{d}\right)dt \end{array}\right]}^{T}\\ y_{0} & =x_{3}. \end{array}\right. \end{array}$$

The subsystems (47) can be rewritten as:

(50) $$\left\{\begin{array}{cc} \underset{x_{0}}{\underset{︸}{\left[\begin{array}{c} {\dot{\theta }}_{e}\\ {\dot{i}}_{q}\\ {\dot{i}}_{d}\\ {\dot{x}}_{31}\\ {\dot{x}}_{32} \end{array}\right]}} & =\underset{A_{0}}{\underset{︸}{\left[\begin{array}{ccccc} 0 & 1 & 1 & 0 & 0\\ 0 & -\frac{\psi_{r}}{L_{q}}-\frac{R_{s}}{L_{q}} & -\frac{\psi_{r}}{L_{q}} & 0 & 0\\ 0 & 0 & -\frac{R_{s}}{L_{d}} & 0 & 0\\ 0 & 1 & 0 & 0 & 0\\ 0 & 0 & 1 & 0 & 0 \end{array}\right]}}\underset{x_{0}}{\underset{︸}{\left[\begin{array}{c} \theta_{e}\\ i_{q}\\ i_{d}\\ x_{31}\\ x_{32} \end{array}\right]}}+\underset{A_{1}}{\underset{︸}{\left[\begin{array}{c} 1\\ -\frac{\psi_{r}}{L_{q}}\\ 0\\ 0\\ 0 \end{array}\right]}}\underset{x_{1}}{\underset{︸}{\omega_{e}}}+\underset{B_{0}}{\underset{︸}{\left[\begin{array}{cc} 0 & 0\\ \frac{1}{L_{q}} & 0\\ 0 & \frac{1}{L_{d}}\\ 0 & 0\\ 0 & 0 \end{array}\right]}}\underset{u}{\underset{︸}{\left[\begin{array}{c} u_{q}\\ u_{d} \end{array}\right]}}+\underset{E_{0}}{\underset{︸}{\left[\begin{array}{cc} 0 & 0\\ 0 & 0\\ 0 & 0\\ 1 & 0\\ 0 & 1 \end{array}\right]}}\underset{f_{s}}{\underset{︸}{\left[\begin{array}{c} f_{q}\\ f_{d} \end{array}\right]}}+\underset{F_{2}}{\underset{︸}{\left[\begin{array}{c} 0\\ -\frac{L_{d}}{L_{q}}\omega_{e}i_{d}\\ \frac{L_{q}}{L_{d}}\omega_{e}i_{q}\\ 0\\ 0 \end{array}\right]}}\\ \underset{y_{0}}{\underset{︸}{\left[\begin{array}{c} x_{31}\\ x_{32} \end{array}\right]}} & =\underset{C_{0}}{\underset{︸}{\left[\begin{array}{ccccc} 0 & 0 & 0 & 1 & 0\\ 0 & 0 & 0 & 0 & 1 \end{array}\right]}}\underset{x_{0}}{\underset{︸}{{\left[\begin{array}{ccccc} \theta_{e} & i_{q} & i_{d} & x_{31} & x_{32} \end{array}\right]}^{T}}}. \end{array}\right.$$

The subsystems (46) can be rewritten as:

(51) $$\left\{\begin{array}{cc} \underset{x_{1}}{\underset{︸}{{\dot{\omega }}_{e}}} & =\underset{A_{11}}{\underset{︸}{\left(-\frac{B}{J}\right)}}\underset{x_{1}}{\underset{︸}{\omega_{e}}}+\underset{A_{2}}{\underset{︸}{\left[\begin{array}{ccccc} 0 & \frac{3n_{p}^{2}}{2J}\psi_{r} & 0 & 0 & 0 \end{array}\right]}}\underset{x_{0}}{\underset{︸}{\left[\begin{array}{c} \theta_{e}\\ i_{q}\\ i_{d}\\ x_{31}\\ x_{32} \end{array}\right]}}+\underset{f_{1}\left(x\right)}{\underset{︸}{\left(\frac{3n_{p}^{2}\left(L_{d}-L_{q}\right)}{2J}i_{d}i_{q}\right)}}+\underset{D_{1}}{\underset{︸}{\left(-\frac{n_{p}}{J}\right)}}\underset{d}{\underset{︸}{T_{L}}}\\ \underset{y_{1}}{\underset{︸}{\omega_{e}}} & =\underset{C_{11}}{\underset{︸}{1}}\cdot \underset{x_{1}}{\underset{︸}{\omega_{e}}}. \end{array}\right.$$

The IPMSM parameters are listed in Table 1.

Substitute the IPMSM parameters into the PMSM-driven system; the new Subsystem (50) can be represented in state-space form as:

(52) $$\left\{\begin{array}{cc} \underset{{\dot{x}}_{0}}{\underset{︸}{\left[\begin{array}{c} {\dot{\theta }}_{e}\\ {\dot{i}}_{q}\\ {\dot{i}}_{d}\\ {\dot{x}}_{31}\\ {\dot{x}}_{32} \end{array}\right]}} & =\underset{A_{0}}{\underset{︸}{\left[\begin{array}{ccccc} 0 & 0 & 0 & 0 & 0\\ 0 & -383.3 & 23.3 & 0 & 0\\ 0 & 0 & -1150 & 0 & 0\\ 0 & 1 & 0 & 0 & 0\\ 0 & 0 & 1 & 0 & 0 \end{array}\right]}}\underset{x_{0}}{\underset{︸}{\left[\begin{array}{c} \theta_{e}\\ i_{q}\\ i_{d}\\ x_{31}\\ x_{32} \end{array}\right]}}+\underset{A_{1}}{\underset{︸}{\left[\begin{array}{c} 1\\ -23.333\\ 0\\ 0\\ 0 \end{array}\right]}}\underset{x_{1}}{\underset{︸}{\omega_{e}}}+\underset{B_{0}}{\underset{︸}{\left[\begin{array}{cc} 0 & 0\\ 133.33 & 0\\ 0 & 400\\ 0 & 0\\ 0 & 0 \end{array}\right]}}\underset{u}{\underset{︸}{\left[\begin{array}{c} u_{q}\\ u_{d} \end{array}\right]}}+\underset{E_{0}}{\underset{︸}{\left[\begin{array}{cc} 0 & 0\\ 0 & 0\\ 0 & 0\\ 1 & 0\\ 0 & 1 \end{array}\right]}}f_{s}+\underset{F_{0}\left(x\right)}{\underset{︸}{\left[\begin{array}{c} 0\\ -\frac{1}{3}\omega_{e}i_{d}\\ 3\omega_{e}i_{q}\\ 0\\ 0 \end{array}\right]}}\\ y_{0} & =\left[\begin{array}{c} y_{01}\\ y_{02} \end{array}\right]=\underset{C_{0}}{\underset{︸}{\left[\begin{array}{ccccc} 0 & 0 & 0 & 1 & 0\\ 0 & 0 & 0 & 0 & 1 \end{array}\right]}}\underset{x_{0}}{\underset{︸}{{\left[\begin{array}{ccccc} \theta_{e} & i_{q} & i_{d} & x_{31} & x_{32} \end{array}\right]}^{T}}}=\underset{x_{3}}{\underset{︸}{{\left[\begin{array}{cc} x_{31} & x_{32} \end{array}\right]}^{T}}}, \end{array}\right.$$

and the new Subsystem (51) can be represented in state-space form as:

(53) $$\left\{\begin{array}{cc} \underset{{\dot{x}}_{1}}{\underset{︸}{{\dot{\omega }}_{e}}} & =\underset{A_{11}}{\underset{︸}{0.125}}\underset{x_{1}}{\underset{︸}{\omega_{e}}}+\underset{A_{2}}{\underset{︸}{\left[\begin{array}{ccccc} 0 & 5250 & 0 & 0 & 0 \end{array}\right]}}\underset{x_{0}}{\underset{︸}{{\left[\begin{array}{ccccc} \theta_{e} & i_{q} & i_{d} & x_{31} & x_{32} \end{array}\right]}^{T}}}+\underset{f_{1}\left(x\right)}{\underset{︸}{\left(-1200i_{d}i_{q}\right)}}-\underset{D_{1}}{\underset{︸}{5000}}\underset{d}{\underset{︸}{T_{L}}}\\ y_{1} & =\underset{x_{1}}{\underset{︸}{\omega_{e}}}. \end{array}\right.$$

The Lipschitz constant of PMSM was chosen to be $\gamma_{1}$ = $\gamma_{2}$ = γ = 0.6 [27]. The LMI toolbox in MATLAB is used to solve the LMI. The following solutions for the SMOs can be computed as:

$P_{1}=\left[0.0140\right]$, $P_{0}=\left[\begin{array}{ccccc} 9.4885 & 0.0373 & 0 & -0.0001 & 0\\ 0.0373 & 0.8029 & 0 & 0.0010 & 0\\ 0 & 0 & 0.2416 & 0 & 0.0001\\ -0.0001 & 0.0010 & 0 & 9.5667 & 0\\ 0 & 0 & 0.0001 & 0 & 9.5667 \end{array}\right]$, $L_{1}=\left[3464\right]$, $L_{0}=\left[\begin{array}{cc} 0.0010 & 0\\ 0.2401 & 0\\ 0 & 0.2185\\ 5.7244 & 0\\ 0 & 5.7243 \end{array}\right]$, $F_{1}=\left[-70.1723\right]$, $F_{0}=\left[\begin{array}{cc} 9.5667 & 0.0000\\ 0.0000 & 9.5667 \end{array}\right]$.

For (53), the SMO as (18) is designed:

(54) $$\left\{\begin{array}{cc} \underset{{\dot{x}}_{1}}{\underset{︸}{{\dot{\hat{\omega }}}_{e}}} & =\underset{A_{11}}{\underset{︸}{0.125}}\underset{{\hat{x}}_{1}}{\underset{︸}{{\hat{\omega }}_{e}}}+\underset{A_{2}}{\underset{︸}{\left[\begin{array}{ccccc} 0 & 5250 & 0 & 0 & 0 \end{array}\right]}}\underset{{\hat{x}}_{0}}{\underset{︸}{{\left[\begin{array}{ccccc} {\hat{\theta }}_{e} & {\hat{i}}_{q} & {\hat{i}}_{d} & {\hat{x}}_{31} & \;{\hat{x}}_{32} \end{array}\right]}^{T}}}+\underset{f_{1}\left(x\right)}{\underset{︸}{\left(-1200{\hat{i}}_{d}{\hat{i}}_{q}\right)}}+\underset{D_{1}}{\underset{︸}{\left(-5000\right)}}w_{1}\\ & +3464(y_{1}-{\hat{y}}_{1})\\ {\hat{y}}_{1} & =\underset{x_{1}}{\underset{︸}{{\hat{\omega }}_{e}}}, \end{array}\right.$$

where $w_{1}$ is the input signal of sliding mode, expressed as:

$$\begin{array}{c} w_{1}=\left\{\begin{array}{ccc} -\rho_{1}\frac{-70.1723({\hat{y}}_{1}-y_{1})}{∥-70.1723({\hat{y}}_{1}-y_{1})∥+\delta } & if & {\hat{y}}_{1}-y_{1}\neq 0\\ 0 & if & {\hat{y}}_{1}-y_{1}=0. \end{array}\right. \end{array}$$

For (52), the SMO as (20) is designed:

(55) $$\begin{array}{c} \left\{\begin{array}{cc} \underset{{\dot{\hat{x}}}_{0}}{\underset{︸}{\left[\begin{array}{c} {\dot{\hat{\theta }}}_{e}\\ {\dot{\hat{i}}}_{q}\\ {\dot{\hat{i}}}_{d}\\ {\dot{\hat{x}}}_{31}\\ {\dot{\hat{x}}}_{32} \end{array}\right]}} & =\underset{A_{0}}{\underset{︸}{\left[\begin{array}{ccccc} 0 & 0 & 0 & 0 & 0\\ 0 & -383.3 & 23.3 & 0 & 0\\ 0 & 0 & -1150 & 0 & 0\\ 0 & 1 & 0 & 0 & 0\\ 0 & 0 & 1 & 0 & 0 \end{array}\right]}}\underset{{\hat{x}}_{0}}{\underset{︸}{\left[\begin{array}{c} {\hat{\theta }}_{e}\\ {\hat{i}}_{q}\\ {\hat{i}}_{d}\\ {\hat{x}}_{31}\\ {\hat{x}}_{32} \end{array}\right]}}+\underset{A_{1}}{\underset{︸}{\left[\begin{array}{c} 1\\ -23.333\\ 0\\ 0\\ 0 \end{array}\right]}}\underset{x_{1}}{\underset{︸}{{\hat{\omega }}_{e}}}+\underset{B_{0}}{\underset{︸}{\left[\begin{array}{cc} 0 & 0\\ 133.33 & 0\\ 0 & 400\\ 0 & 0\\ 0 & 0 \end{array}\right]}}\underset{u}{\underset{︸}{\left[\begin{array}{c} u_{q}\\ u_{d} \end{array}\right]}}\\ & +\underset{E_{0}}{\underset{︸}{\left[\begin{array}{cc} 0 & 0\\ 0 & 0\\ 0 & 0\\ 1 & 0\\ 0 & 1 \end{array}\right]}}w_{0}+\underset{F_{0}\left(\hat{x}\right)}{\underset{︸}{\left[\begin{array}{c} 0\\ -\frac{1}{3}{\hat{\omega }}_{e}{\hat{i}}_{d}\\ 3{\hat{\omega }}_{e}{\hat{i}}_{q}\\ 0\\ 0 \end{array}\right]}}+\underset{L_{0}}{\underset{︸}{\left[\begin{array}{cc} 0.0010 & 0\\ 0.2401 & 0\\ 0 & 0.2185\\ 5.7244 & 0\\ 0 & 5.7243 \end{array}\right]}}\left(y_{0}-{\hat{y}}_{0}\right)\\ {\hat{y}}_{0} & =\left[\begin{array}{c} {\hat{y}}_{01}\\ {\hat{y}}_{02} \end{array}\right]=\underset{C_{0}}{\underset{︸}{\left[\begin{array}{ccccc} 0 & 0 & 0 & 1 & 0\\ 0 & 0 & 0 & 0 & 1 \end{array}\right]}}\underset{{\hat{x}}_{0}}{\underset{︸}{{\left[\begin{array}{ccccc} {\hat{\theta }}_{e} & {\hat{i}}_{q} & {\hat{i}}_{d} & {\hat{x}}_{31} & {\hat{x}}_{32} \end{array}\right]}^{T}}}=\underset{{\hat{x}}_{3}}{\underset{︸}{{\left[\begin{array}{cc} {\hat{x}}_{31} & {\hat{x}}_{32} \end{array}\right]}^{T}}}, \end{array}\right. \end{array}$$

where $w_{0}$ is the input signal of sliding mode, expressed as:

$$\begin{array}{c} w_{0}=\left\{\begin{array}{ccc} -\rho_{0}\frac{\left[\begin{array}{cc} 9.5667 & 0.0000\\ 0.0000 & 9.5667 \end{array}\right]\left({\hat{y}}_{0}-y_{0}\right)}{∥\left[\begin{array}{cc} 9.5667 & 0.0000\\ 0.0000 & 9.5667 \end{array}\right]({\hat{y}}_{0}-y_{0}∥+\delta } & if & {\hat{y}}_{0}-y_{0}\neq 0\\ 0 & if & {\hat{y}}_{0}-y_{0}=0. \end{array}\right. \end{array}$$

Select $\rho_{1}$ = 100, $\rho_{0}=\left[\begin{array}{cc} 10000 & 0\\ 0 & 10000 \end{array}\right]$ and δ = 0.01, to complete the SMO design.

## 5. Simulations and Experiments

**Figure 1:**
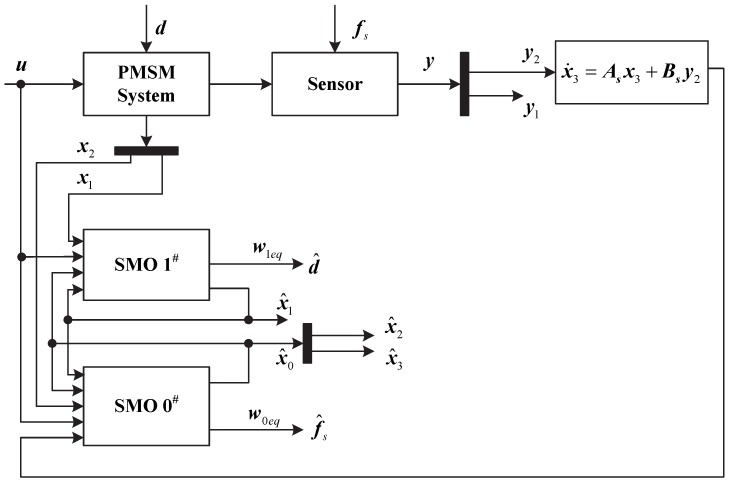
Schematic of the sensor fault reconstruction and unknown input disturbances estimation by SMO.

To check the performance of the proposed scheme, simulations are performed on MATLAB/Simulink. The complete sliding mode observer-based current sensor fault reconstruction and unknown load estimation scheme is shown in Figure 1.

### 5.1. Simulation Results

The initial rotor electrical angular velocity is set to 300 rad/s. The load torque is set as 2 Nm. The $i_{d}=0$ control scheme is carried out on an IPMSM.

#### 5.1.1. Case 1: Incipient Fault of Current Sensor

In the case of incipient faults of the *d*-axis and *q*-axis current sensor, the faults are expressed as follows [28]:

(56) $$\begin{array}{c} \begin{array}{c} f_{q1}\left(t\right)=\left\{\begin{array}{c} 0\\ 2\exp(0.0667t) \end{array}\right.\begin{array}{c} t<0.5s\\ t\geq s \end{array},f_{d1}\left(t\right)=\left\{\begin{array}{c} 0\\ \tanh(t) \end{array}\begin{array}{c} t<0.5s\\ t\geq 0.5s \end{array}\right. \end{array} \end{array}$$

**Figure 2:**
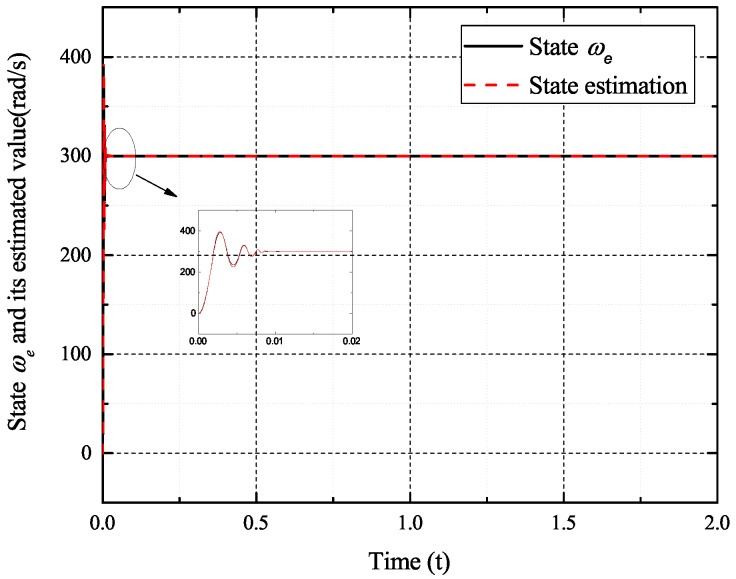
State $\omega_{e}$ and its estimated value ${\hat{\omega }}_{e}$.

**Figure 3:**
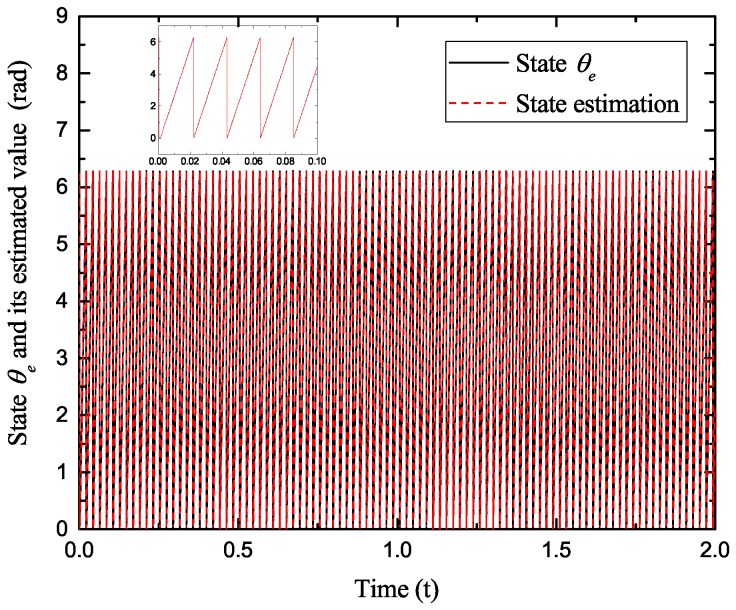
State $\theta_{e}$ and its estimated value ${\hat{\theta }}_{e}$.

**Figure 4:**
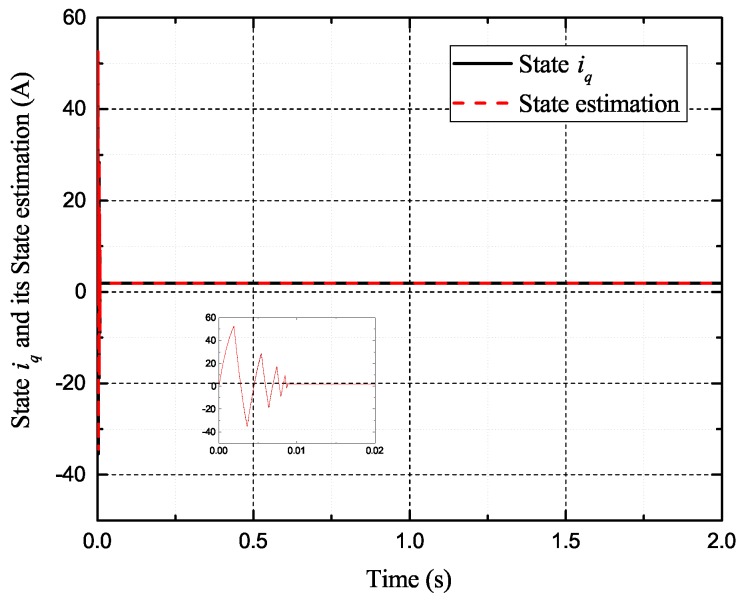
State $i_{q}$ and its estimated value ${\hat{i}}_{q}$.

**Figure 5:**
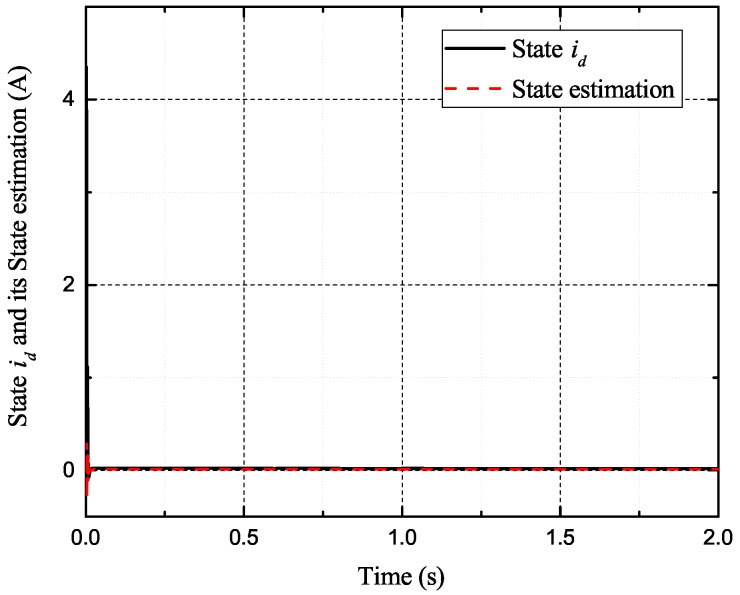
State $i_{d}$ and its estimated value ${\hat{i}}_{d}$.

**Figure 6:**
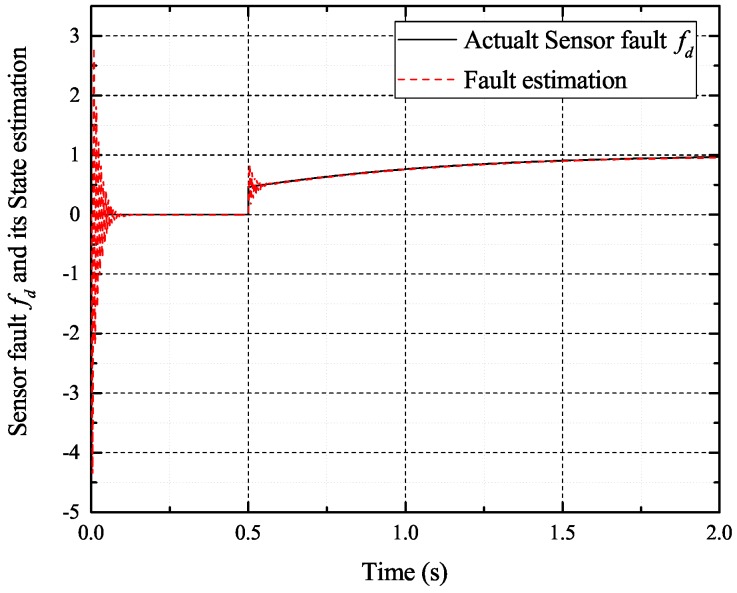
Sensor fault $f_{d}$ and its estimated value ${\hat{f}}_{d}$.

**Figure 7:**
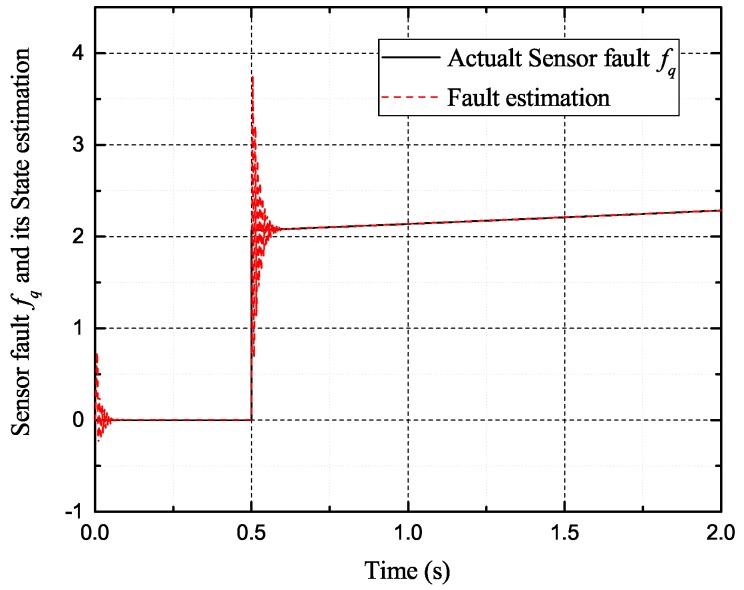
Sensor fault $f_{q}$ and its estimated value ${\hat{f}}_{q}$.

**Figure 8:**
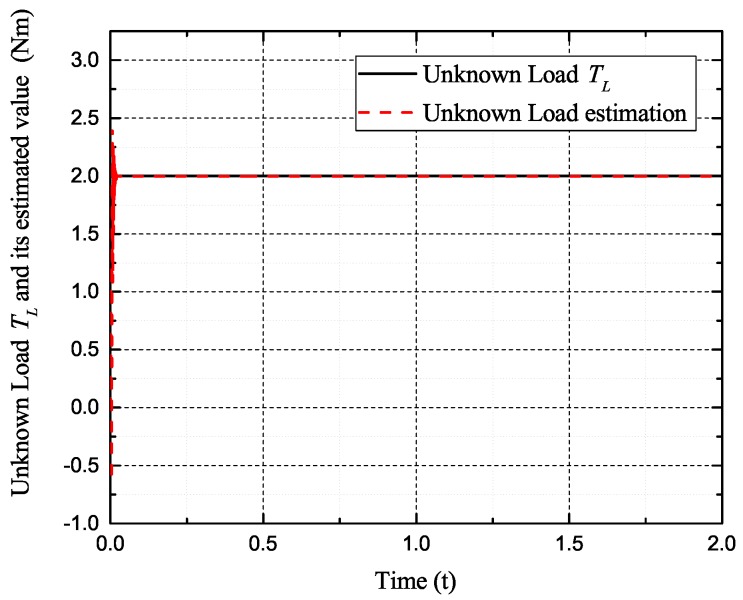
Unknown load disturbances $T_{L}$ and its estimated value ${\hat{T}}_{L}$.

Figure 2, Figure 3, Figure 4 and Figure 5 exhibit the states and their estimated values, respectively. Figure 6 and Figure 7 show the *d*-*q* axis sensor incipient faults and their estimated trajectories, respectively. Figure 8 shows the unknown load disturbances and its estimated trajectories. It can be seen from the figures that both *d*-*q* axis current sensor incipient faults and unknown input load disturbances can be accurately reconstructed in the PMSM-driven system.

#### 5.1.2. Case 2: Intermittent Fault of Current Sensor

In the case of intermittent faults of the *d*-axis and *q*-axis current sensor, the faults are expressed as follows:

(57) $$\begin{array}{c} f_{q2}\left(t\right)=\left\{\begin{array}{cc} 0 & t<0.5s\\ 0.5 & 0.5s\leq t<0.8s\\ 0 & 0.8s\leq t<1s\\ 0.8 & 1s\leq t<1.2s\\ 0.2 & 1.2s\leq t<1.4s\\ \begin{array}{c} 0.5\\ 0 \end{array} & \begin{array}{c} 1.4s\leq t<1.6s\\ t\geq 1.6s \end{array} \end{array}\right.\begin{array}{c} , \end{array}f_{d2}\left(t\right)=\left\{\begin{array}{cc} 0 & t<0.6s\\ 1 & 0.6s\leq t<1s\\ 0 & 1s\leq t<1.2s\\ 0.8 & 1.2s\leq t<1.4s\\ 0.2 & 1.4s\leq t<1.6s\\ 0 & t\geq 1.6s \end{array}\right. \end{array}$$

**Figure 9:**
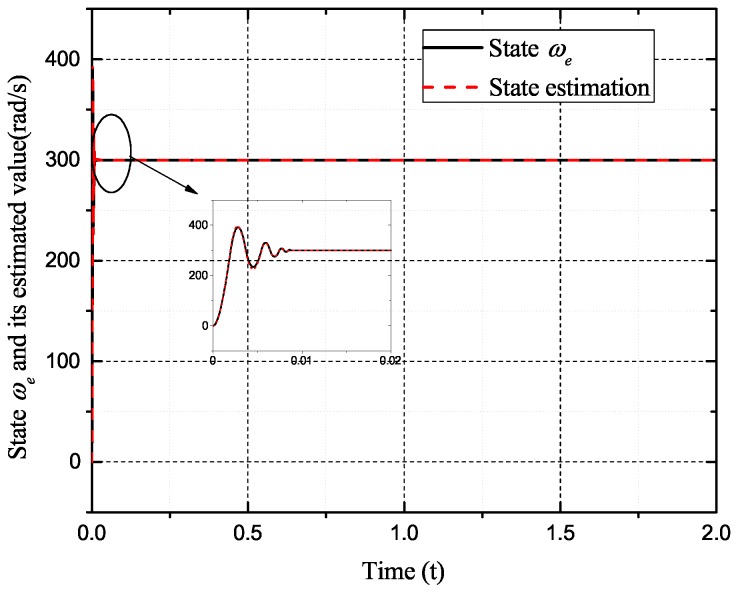
State $\omega_{e}$ and its estimated value ${\hat{\omega }}_{e}$.

**Figure 10:**
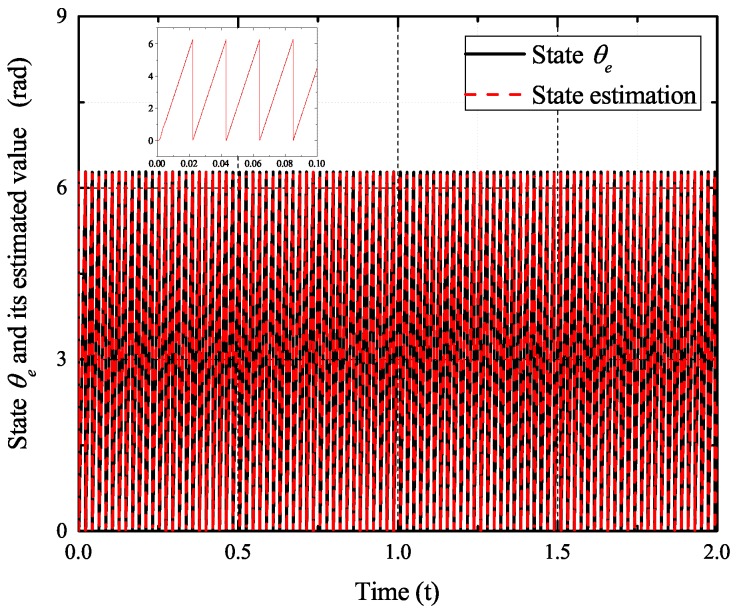
State $\theta_{e}$ and its estimated value ${\hat{\theta }}_{e}$.

**Figure 11:**
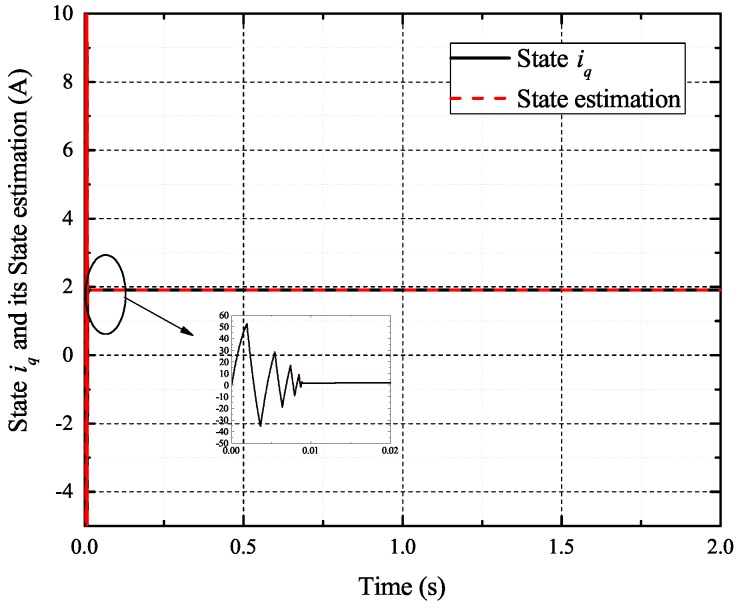
State $i_{q}$ and its estimated value ${\hat{i}}_{q}$.

**Figure 12:**
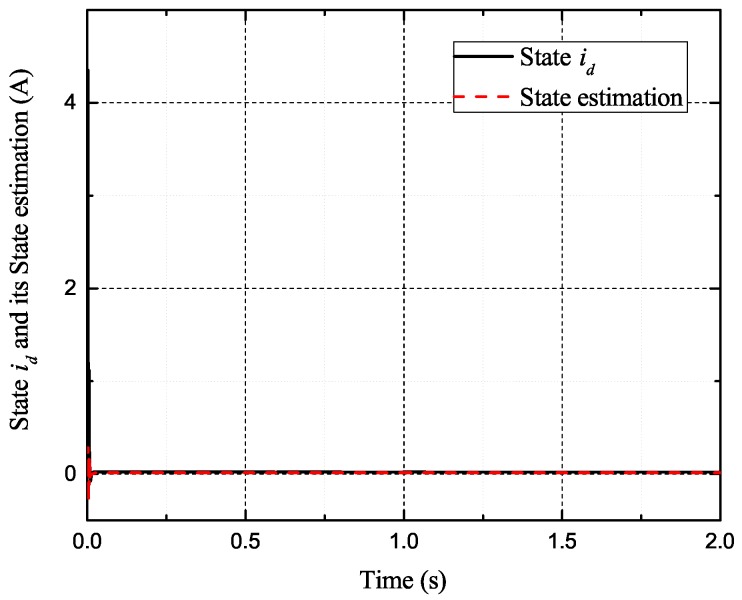
State $i_{d}$ and its estimated value ${\hat{i}}_{d}$.

**Figure 13:**
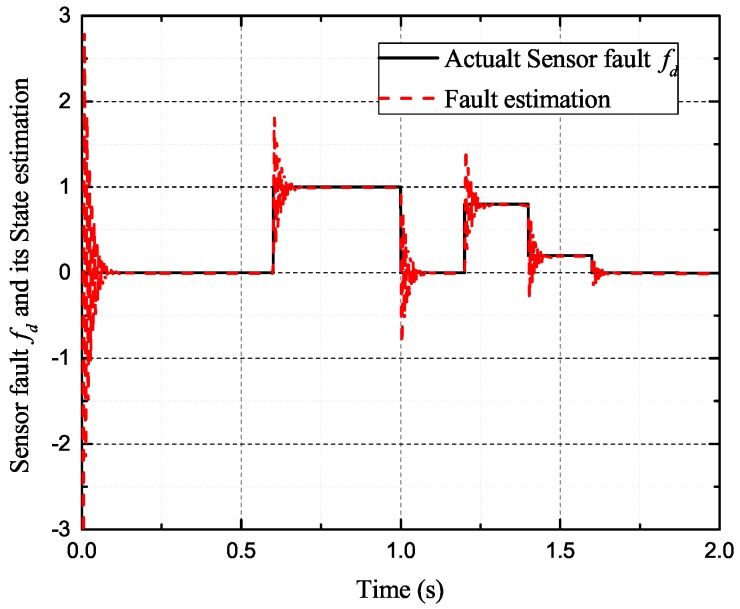
Sensor fault $f_{d}$ and its estimated value ${\hat{f}}_{d}$.

**Figure 14:**
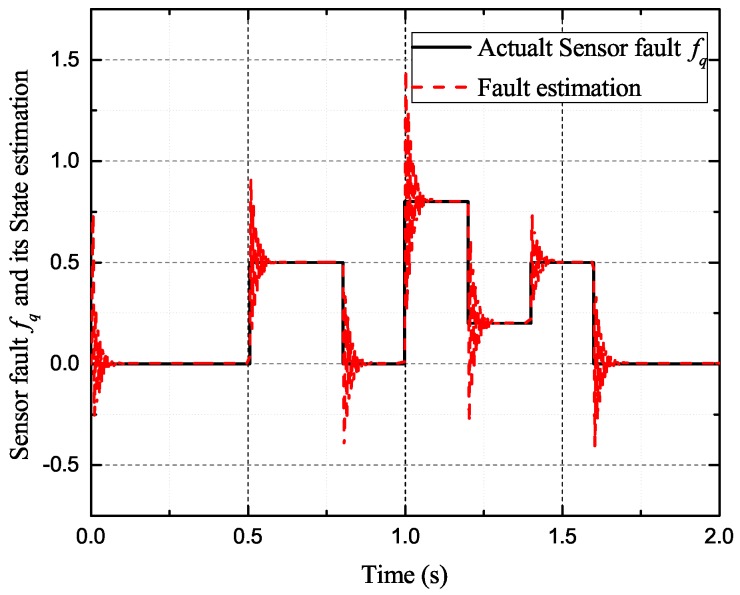
Sensor fault $f_{q}$ and its estimated value ${\hat{f}}_{q}$.

**Figure 15:**
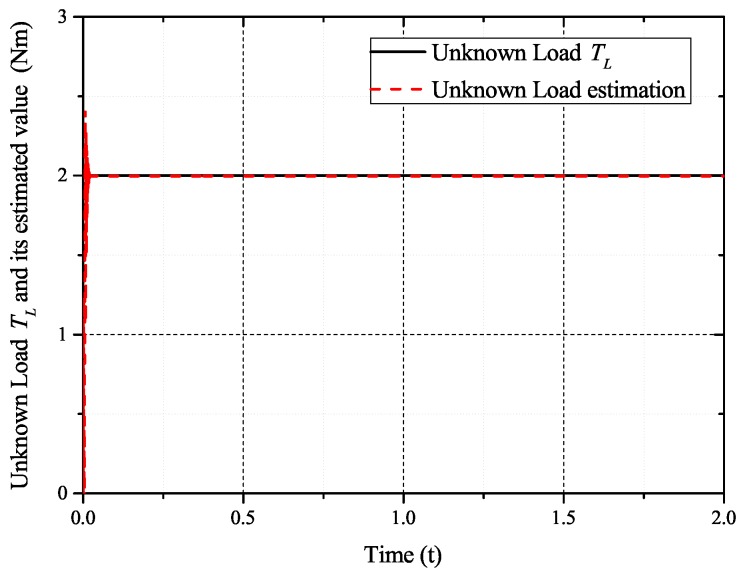
Unknown load disturbances $T_{L}$ and its estimated value ${\hat{T}}_{L}$.

Figure 9, Figure 10, Figure 11 and Figure 12 exhibit the states and their estimated values, respectively. Figure 13 and Figure 14 show the *d*-*q* axis sensor intermittent faults and their estimated trajectories, respectively. Figure 15 shows the unknown input load disturbances and its estimated trajectories. It can be seen from the figures that both *d*-*q* axis current sensor intermittent faults and unknown input load disturbances can be accurately reconstructed in the PMSM-driven system.

#### 5.1.3. Case 3: High Frequency and Low Frequency Fault of Current Sensor

In the case of the low frequency of the *d*-axis current sensor and the high frequency fault of the *q*-axis, the faults are expressed as follows [29]:

(58) $$\begin{array}{c} f_{q3}\left(t\right)=\left\{\begin{array}{cc} 0 & t<0.5s\\ \left(0.5\sin\left(15t\right)+0.25\sin\left(10t\right)\right)\frac{0.2{∥y∥}_{2}^{2}}{{∥y∥}_{2}+0.5} & t\geq 0.5s \end{array}\right.\\ f_{d3}\left(t\right)=\left\{\begin{array}{c} 0\\ \sin(0.5t)+0.2\sin(2t) \end{array}\right.\begin{array}{c} t<0.6s\\ t\geq 0.6s \end{array} \end{array}$$

**Figure 16:**
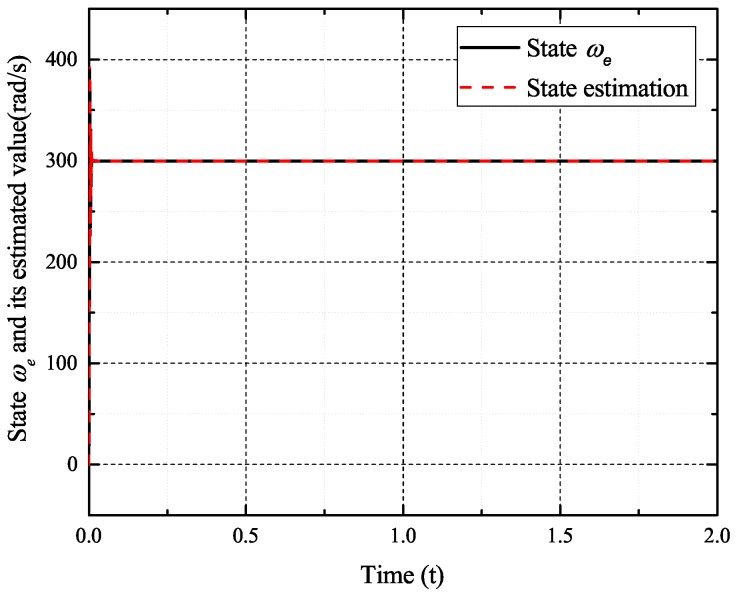
State $\omega_{e}$ and its estimated value ${\hat{\omega }}_{e}$.

**Figure 17:**
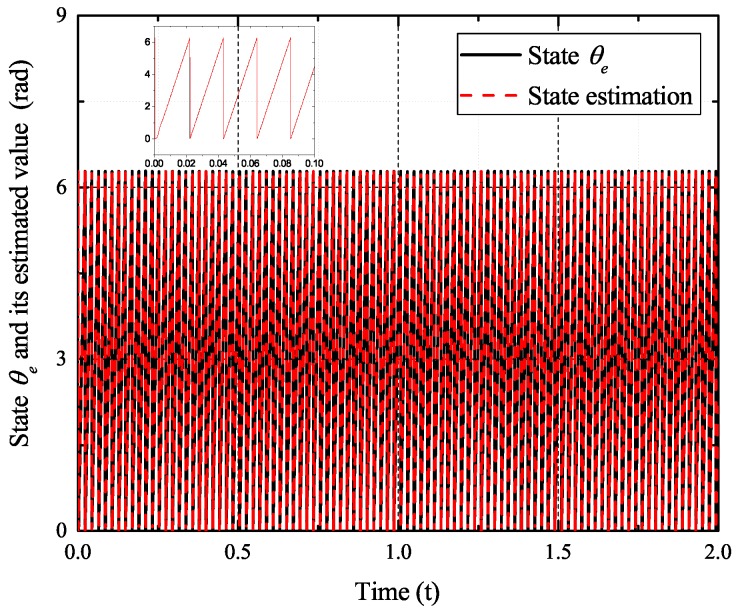
State $\theta_{e}$ and its estimated value ${\hat{\theta }}_{e}$.

**Figure 18:**
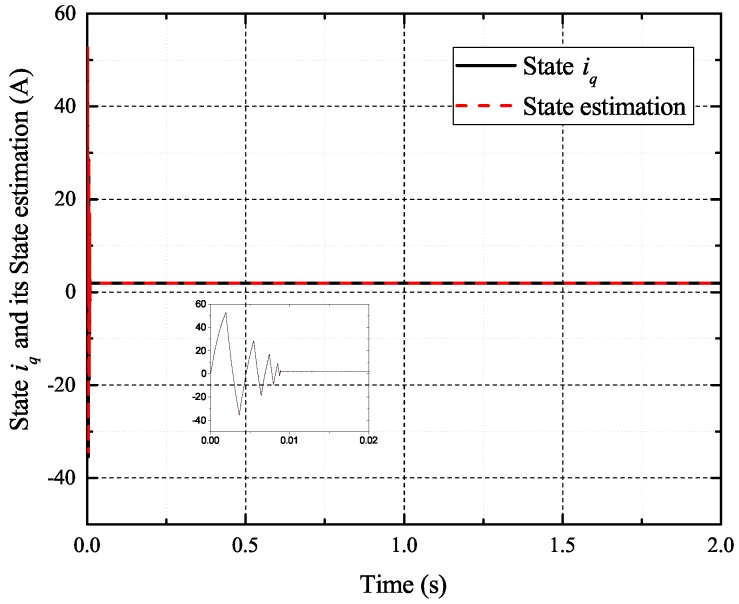
State $i_{q}$ and its estimated value ${\hat{i}}_{q}$.

**Figure 19:**
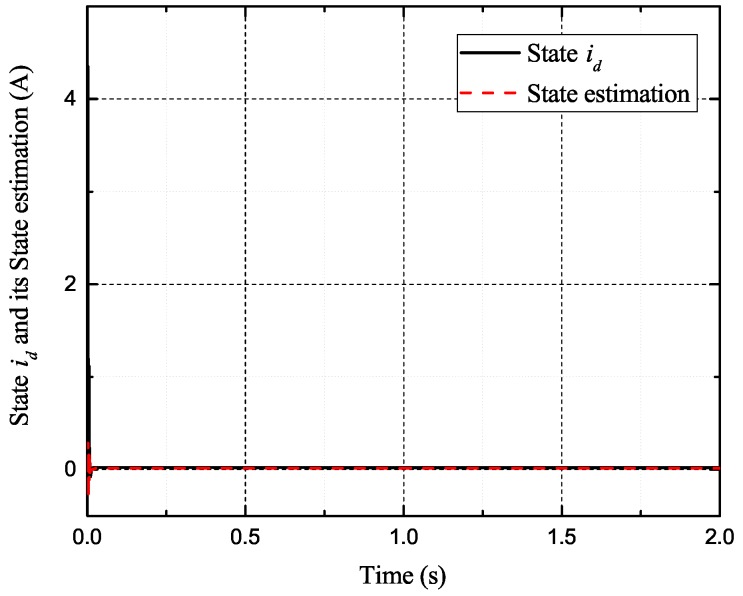
State $i_{d}$ and its estimated value ${\hat{i}}_{d}$.

**Figure 20:**
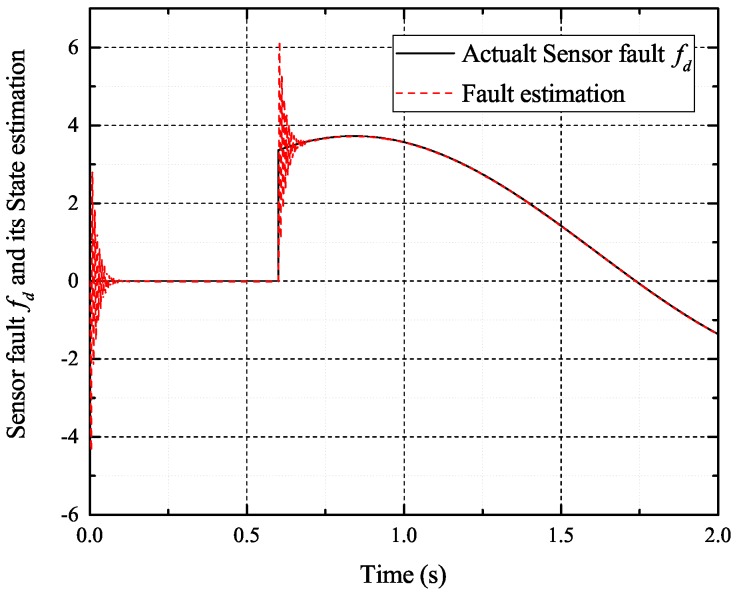
Sensor fault $f_{d}$ and its estimated value ${\hat{f}}_{d}$.

**Figure 21:**
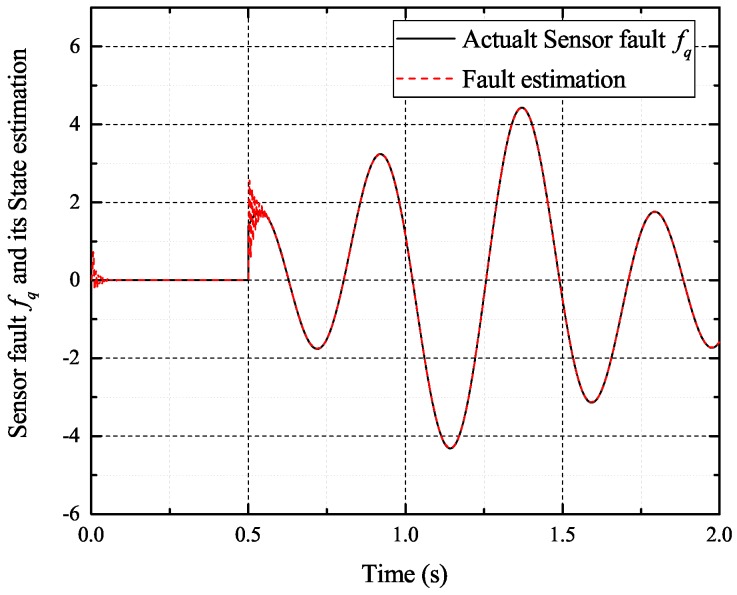
Sensor fault $f_{q}$ and its estimated value ${\hat{f}}_{q}$.

**Figure 22:**
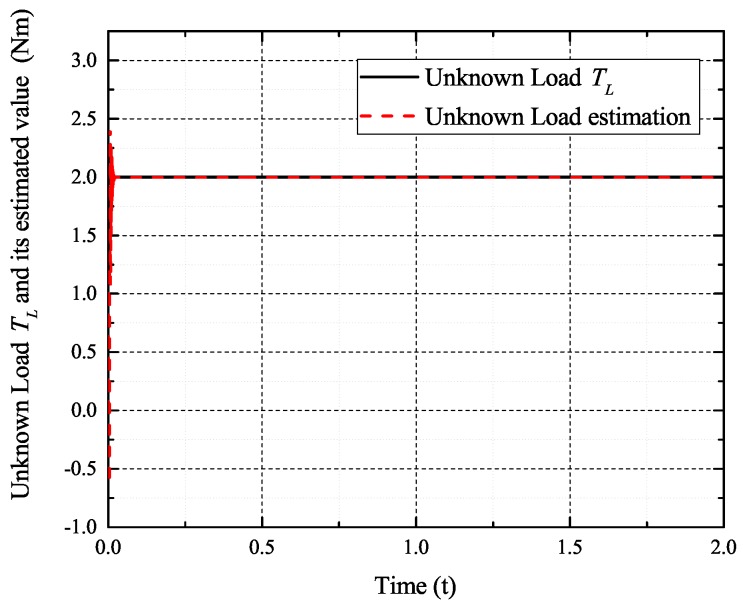
unknown load disturbances $T_{L}$ and its estimated value ${\hat{T}}_{L}$.

Figure 16, Figure 17, Figure 18 and Figure 19 exhibit the states and their estimated values, respectively. Figure 20 and Figure 21 show the *d*-*q* axis sensor high and low frequency faults and their estimated trajectories, respectively. Figure 22 shows the unknown input load disturbances and its estimated trajectories. It can be seen from the figures that both *d*-*q* axis current sensor high and low frequency faults and unknown input load disturbances can be accurately reconstructed in the PMSM-driven system.

### 5.2. Experiments Results

RT-LAB is a modular, distributed, real-time platform. It supports model-based design using rapid control prototyping (RCP) and hardware-in-the-loop simulation (HILS) for complex dynamic systems [30].

**Figure 23:**
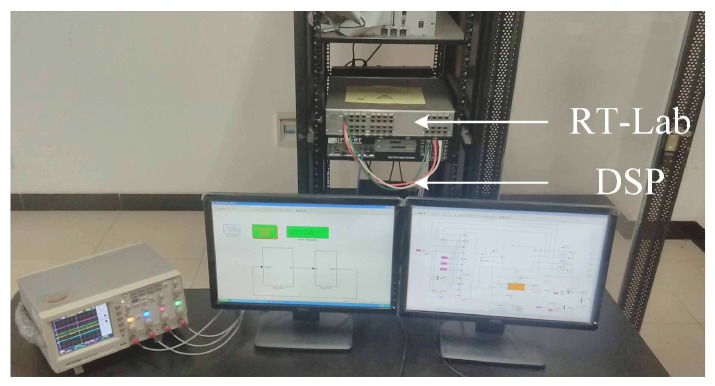
RT-LAB platform.

**Figure 24:**
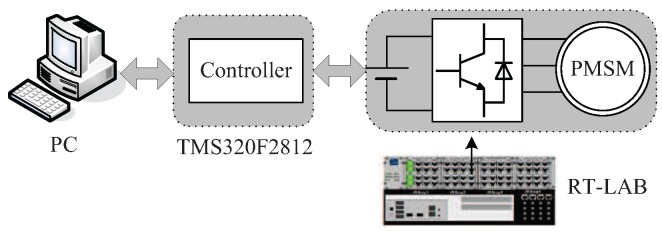
Configuration of the RT-LAB hardware-in-the-loop simulation (HILS) system.

To implement the proposed scheme, HILS experiments are carried out on an OP5600 RT-LAB platform. The RT-LAB platform is shown in Figure 23, and the configuration is shown in Figure 24. The controller is a TMS320F2812 digital signal processor, which implements high-performance control and computation. The inverter, PMSM system and current sensor faults are simulated by RT-LAB. The PWM switching frequency is chosen as 5 kHz. The sampling period is chosen as 20 μs.

#### 5.2.1. Case 1: Incipient Faults of Current Sensor

**Figure 25:**
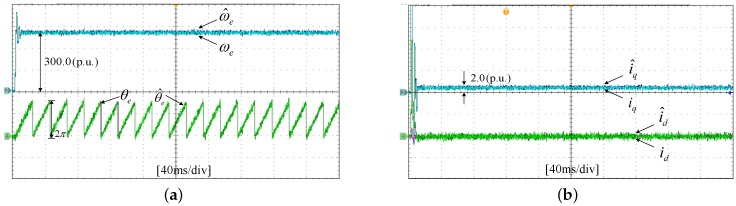
Experimental results of states and their estimated values. (**a**) States $\omega_{e}$, $\theta_{e}$ and their estimated value ${\hat{\omega }}_{e}$, ${\hat{\theta }}_{e}$; (**b**) states $i_{d}$, $i_{q}$ and their estimated value ${\hat{i}}_{d}$, ${\hat{i}}_{q}$.

**Figure 26:**
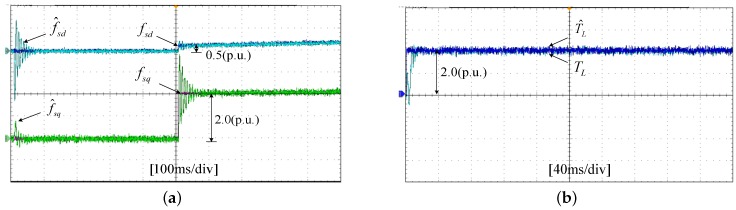
Experimental results of incipient sensor faults, unknown load and their estimated values. (**a**) Sensor faults $f_{d}$, $f_{q}$ and their estimated values ${\hat{f}}_{d}$, ${\hat{f}}_{q}$; (**b**) unknown load $T_{L}$ and its estimated value ${\hat{T}}_{L}$.

The experiments of the *d*-*q* axis current sensor incipient faults Equation (56) are shown in Figure 25 and Figure 26. Figure 25 shows the states and their estimated values, respectively. Figure 26 exhibits the *d*-*q* axis sensor incipient faults and their estimated trajectories, the unknown load disturbances and its estimated trajectories, respectively.

It can be seen from the figures that both the states, *d*-*q* axis current sensor incipient faults and unknown input load disturbances can be accurately reconstructed and estimated by SMOs in PMSM driven system.

#### 5.2.2. Case 2: Intermittent Fault of Current Sensor

**Figure 27:**
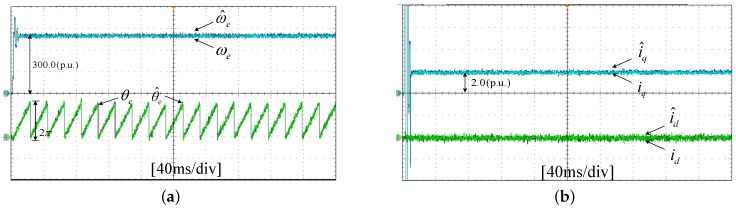
Experimental results of states and their estimated values. (**a**) States $\omega_{e}$, $\theta_{e}$ and their estimated values ${\hat{\omega }}_{e}$, ${\hat{\theta }}_{e}$; (**b**) states $i_{d}$, $i_{q}$ and their estimated values ${\hat{i}}_{d}$, ${\hat{i}}_{q}$.

**Figure 28:**
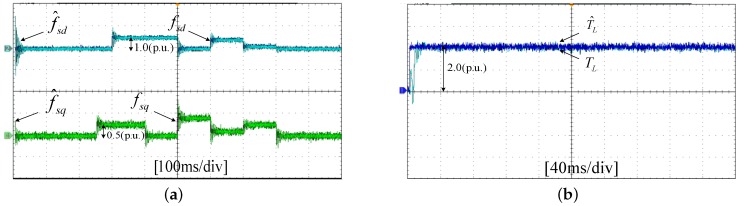
Experimental results of intermittent sensor faults, unknown load and their estimated values. (**a**) Sensor faults $f_{d}$, $f_{q}$ and their estimated values ${\hat{f}}_{d}$, ${\hat{f}}_{q}$; (**b**) unknown load $T_{L}$ and its estimated value ${\hat{T}}_{L}$.

The experiments of *d*-*q* axis current sensor intermittent faults Equation (57) are shown in Figure 27 and Figure 28.

Figure 27 shows the states and their estimated value, respectively. Figure 28 exhibits the *d*-*q* axis sensor intermittent faults and their estimated trajectories, the unknown load disturbances and their estimated trajectories, respectively.

It can be seen from the figures that both states, *d*-*q* axis current sensor intermittent faults and unknown input load disturbances, can be accurately reconstructed and estimated by SMOs in the PMSM-driven system.

#### 5.2.3. Case 3: High Frequency and Low Frequency Fault of Current Sensor

**Figure 29:**
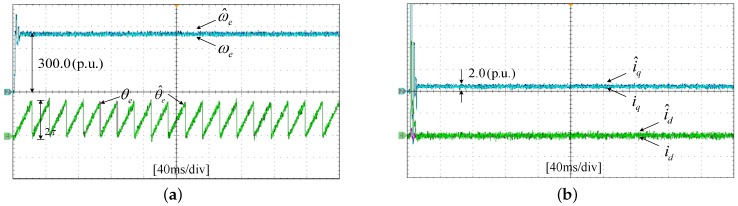
Experimental results of states and their estimated values. (**a**) States $\omega_{e}$, $\theta_{e}$ and their estimated values ${\hat{\omega }}_{e}$, ${\hat{\theta }}_{e}$; (**b**) states $i_{d}$, $i_{q}$ and their estimated values ${\hat{i}}_{d}$, ${\hat{i}}_{q}$.

**Figure 30:**
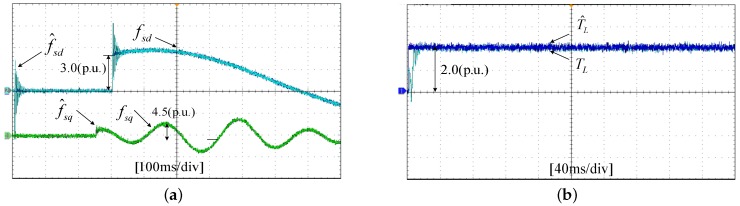
Experimental results of high and low frequency sensor faults, unknown load and their estimated values. (**a**) Sensor faults $f_{d}$, $f_{q}$ and their estimated values ${\hat{f}}_{d}$, ${\hat{f}}_{q}$; (**b**) unknown load $T_{L}$ and its estimated value ${\hat{T}}_{L}$.

The experiments of the *d*-*q* axis current sensor high frequency and low frequency faults Equation (58) are shown in Figure 29 and Figure 30. Figure 29 shows the states and their estimated values, respectively. Figure 30 exhibits the *d*-*q* axis sensor high frequency and low frequency faults and their estimated trajectories, the unknown load disturbances and their estimated trajectories, respectively.

It can be seen from the figures that both states, *d*-*q* axis current sensor high frequency and low frequency faults and unknown input load disturbances, can be accurately reconstructed and estimated by SMOs in the PMSM-driven system.

## 6. Conclusions

This paper proposes a new scheme of reconstructing current sensor faults and estimating unknown load disturbance for permanent magnet synchronous motor (PMSM)-driven systems. The PMSM dynamic mathematical model is transformed into two subsystems; the first subsystem has unknown load disturbance without sensor faults, and the second subsystem has sensor faults without disturbances. Introducing a new state variable, the augmented subsystem, which has sensor faults, can be transformed from having sensor faults to having actuator faults. Then, two SMOs are designed: the unknown load disturbance is estimated by the first SMO, and the sensor faults can be reconstructed by the second SMO. The sufficient conditions for the stability of the proposed scheme are given and expressed as linear matrix inequalities (LMI). The scheme is capable pf estimating the PMSM system states, such as electrical angle, electrical angular velocity and *d*-*q* currents, the load torque and the sensor current faults. The scheme is applicable to incipient fault, intermittent fault, high frequency and low frequency fault, or any other type of fault. The good results of simulation and experiment demonstrate that the proposed scheme can reconstruct current sensor faults and estimate unknown load disturbance for PMSM-driven systems. In the future, the adaptive sliding mode observer-based current sensor fault reconstruction and unknown load disturbance estimation will be designed for the PMSM-driven system.

## Figures and Tables

**Table 1:** Parameters of the permanent magnet synchronous motor.

Parameters	Unit	Values
stator resistance ($R_{s}$)	Ω	2.875
number of pole pairs ($n_{p}$)	pairs	4
*q*-axis inductance ($L_{q}$)	H	0.0075
*d*-axis inductance ($L_{d}$)	H	0.0025
rotor PM flux ($\psi_{r}$)	Wb	0.175
rotational inertia (*J*)	kg·m${}^{2}$	0.0008
viscous friction coefficient (*B*)	Nm·s/rad	0.0001
